# Steering the Clinical Translation of Delivery Systems for Drugs and Health Products

**DOI:** 10.3390/pharmaceutics12040350

**Published:** 2020-04-13

**Authors:** Rosario Pignatello, Pietro Matricardi

**Affiliations:** 1 Department of Drug Sciences, University of Catania, 95125 Catania, Italy; r.pignatello@unict.it; 2 Department of Drug Chemistry and Technologies, Sapienza University of Roma, 00185 Roma, Italy

**Keywords:** drug delivery, controlled release, clinical trials, bioinformatics, medical devices, nutraceuticals

## Abstract

Besides the feasibility for industrial scale-up, accelerating the translation from bench to bedside of new technological strategies for controlled delivery and targeting of drugs and other actives relevant for health management, such as medical devices and nutraceuticals, would benefit from an even earlier evaluation in pre-clinical models and clinical settings. At the same time, translational medicine also performs in the opposite direction, incorporating clinical needs and observations into scientific hypotheses and innovative technological proposals. With these aims, the sessions proposed for the 2019 CRS Italy Chapter Workshop will introduce the experience of Italian and worldwide researchers on how to foster the actual work in controlled release and drug delivery towards a reliable pre-clinical and clinical assessment.

## 1. Aim and Scope of the Meeting

The 2019 Controlled Release Society (CRS) Italy Chapter Annual Workshop was held in Catania on 7–9 November 2019. The University of Catania kindly supported the Workshop offering two wonderful venues: the conference room at “Città della Scienza”, a newly renewed space dedicated to science promotion, formerly an industrial site (http://www.cds.unict.it/) and the main hall of the wonderful “Monastero dei Benedettini Cassinesi” (http://www.monasterodeibenedettini.it/), both in Catania.

The workshop theme was “Steering the Clinical Translation of Delivery Systems for Drugs and Health Products”. The feasibility for industrial scale-up and the translation from bench to bedside of new technological strategies for controlled delivery and targeting of drugs, would benefit from an even earlier evaluation in pre-clinical models and clinical settings. At the same time, translational medicine also performs in the opposite direction, incorporating clinical needs and observations into scientific hypotheses and innovative technological proposals. With these aims, the four sessions in which the 2019 CRS Italy Chapter Workshop was articulated gathered the experience of Italian and worldwide researchers on how to foster the actual work towards a reliable pre-clinical and clinical assessment. Several colleagues from Europe and abroad lectured and stimulated the discussion on exploring new frontiers and tackling new horizons. To foster the discussion on recent research and networking opportunities among attendees, all poster presenters were provided with the opportunity to give a short talk (10 min, in front of the poster).

Over 100 delegates from universities and pharmaceutical companies, mainly from Italy, attended the workshop, which featured 15 speakers with diverse research interests and backgrounds. A summary of the presentations of the invited speakers and the abstracts of the posters are reported hereafter.

## 2. Lectures

### 2.1. Session A: How Informatics Can Support Drug Delivery Technologies

#### 2.1.1. Modeling and Simulation, a Powerful Tool to Inform Drug Development: A First-Time-in-Human Study Design in Oncology

MagniPaoloDepartment of Electrical, Computer and Biomedical Engineering, Università degli Studi di Pavia, 27100 Pavia, Italy; paolo.magni@unipv.it

Mathematical modeling and computer simulations are a powerful tool useful in all the phases of drug development. Mathematical modeling plays a central role in knowledge elicitation and in making explicit all assumptions; computer simulations allow exploration of different scenarios before performing experiments, helping also in designing them and limiting the trial-and-error approach typical of experimental and observational sciences. In this talk, as an example, modeling and simulation is applied to support a first-time-in-human study design in oncology. Data from pharmacokinetic and toxicokinetic studies in monkey and in vivo tumor inhibition growth experiments in xenograft mice were exploited to inform this step by using in the pre-clinical species modeling approaches that are considered reference in the field and then translating them to human [1,2].

First, pre-clinical data were modeled: (i) a 2-compartment population model with linear and saturable elimination from the central compartment was adopted to describe pharmacokinetic (PK) data in monkey; (ii) myelosuppression in monkey was well modeled by the population Friberg model [3] that adequately described the observed neutropenia after single and repeat doses; (iii) antitumor drug efficacy was assessed in xenograft mice by the Simeoni TGI model [4], from which the minimal “effective” concentration for tumor eradication was derived [5].

Then, pre-clinical models were translated to human under clearly defined hypothesis. PK in human was translated from the monkey model by applying the allometric scaling approach; distribution volume and both linear and non-linear clearance were scaled based on body weight using an allometric exponent of 1. Myelosuppression in human caused by the anticancer treatment was predicted by translating the Friberg model previously identified in monkey: time-courses of neutropenia in patients were predicted based on expected PK in human, typical human system-related parameters reported in the literature and drug-effect parameters estimated in monkey [6]. The target exposure in human was obtained from the minimal “effective” concentration derived in mice. The risk–benefit profile of different dosing schedules was evaluated based on predicted neutropenia and expected antitumor activity in patients.

Finally, clinical trial simulations were performed to explore plausible scenarios for the FIH study, enabling the computation of several quantities of interest that supported, for example, the feasibility of the proposed accelerated titration design with 100% dose escalations that reverts to a more conservative design with smaller dose escalations once Grade ≥2 toxicity is observed.

#### 2.1.2. How Computer Science and Biomedical Engineering can Help Drug Discovery Process: In Silico Trials

PappalardoFrancesco*RussoGiuliaDepartment of Drug Sciences, University of Catania, 95125 Catania, Italy*Correspondence: francesco.pappalardo@unict.it

In Silico Trials (IST) represent an innovative application of Virtual Human technology helpful in assisting and supporting the refinement, the reduction, or the replacement of pre-clinical and clinical trials. In this multifaceted challenge perspective, the regulatory authorities are faced with an increasing number of projects developing and applying ISTs ranging from validating ongoing in silico models of specific pathophysiology or applied virtual populations, via technological and infrastructural demands. The last few years have been characterized by an intense activity around so-called regulatory science, aimed to ensure a robust approach to assess the credibility of individual in silico methods as sources of regulatory evidence.

In the regulatory field, the term qualification indicates the overall process that a regulator uses to establish the credibility of a novel method. This process is not entirely codified yet, although some aspects of it are addressed by the ASME V&V-40 standard for medical devices, and in other regulatory documents for drugs.

**Figure 1:**
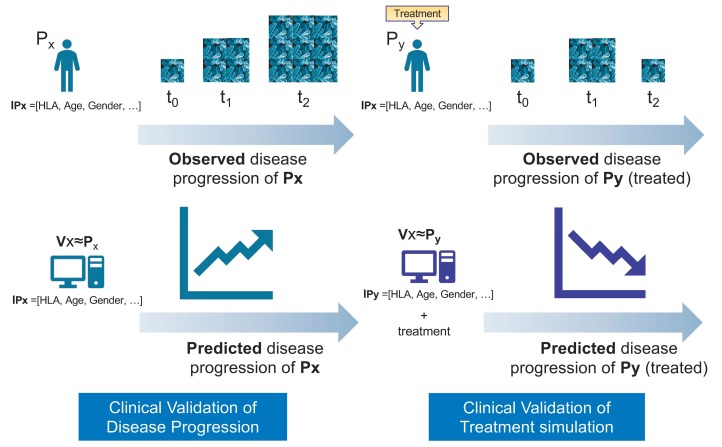
Clinical validation: disease + intervention. Adapted from [7].

Credibility analysis consists of Technical Validation, which is developed within the traditional framework of Verification, Validation, and Uncertainty Quantification (VV&UQ) already well established for other industrial sectors, and Clinical Validation, where the principles of general biomarkers qualification are followed (Figure 1).

#### 2.1.3. Intricate Journey of Micro- and Nano-Carriers for Drug Delivery in the Blood Stream

RackKathrin^1^HooreMasoud^2^GompperGerhard^2^FedosovDmitry A.^2^*1Simulation and Software Technology, German Aerospace Center, 51147 Köln, Germany2Institute of Complex Systems and Institute for Advanced Simulation, Forschungszentrum Jülich 52425 Jülich, Germany*Correspondence: d.fedosov@fz-juelich.de

**Figure 2:**
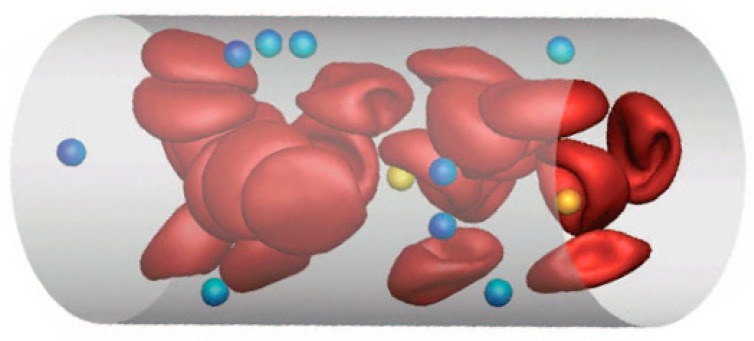
Three-dimensional simulation of blood flow in a tube with several spherical drug carriers.

Drug delivery by various micro- and nanocarriers offers the possibility of controlled transport of pharmaceuticals to targeted sites (e.g., cancerous tissue). The adhesion of micro- and nanocarriers in blood flow is strongly affected by their distribution within the vessel cross-section (Figure 2). To investigate the adhesion potential of carriers of different sizes, we employ mesoscopic hydrodynamic simulations of blood flow to predict margination of carriers or their migration properties toward vessel walls. The margination of carriers is studied for a wide range of hematocrit values and flow rates, and various sizes, ranging from about hundred nanometers to several micrometers, are considered [8].

Our results show that margination strongly depends on the thickness of the available free space close to the wall, the so-called red blood cell-free layer (RBC-FL), in comparison to the carrier size [9]. The carriers with a few micrometers in size are comparable with the RBC-FL thickness and marginate better than their sub-micrometer counterparts. Deformable carriers, in general, show worse margination properties than rigid particles. Particle margination is also found to be most pronounced in small channels with a characteristic size comparable to blood capillaries.

As a conclusion, micron-size particles seem to be favorable for vascular drug delivery in comparison to sub-micron carriers.

#### 2.1.4. Multiscale Modeling and Simulations at the Nanometer Scale for the Optimization of Delivery Systems

PannuzzoMartina*HortaBruno A.C.PrimaveraRositaFrancescoMartina DiRosaCarmelo LaMoghimiSeyed MoeinDecuzziPaoloLaboratory of Nanotechnology for Precision Medicine, Fondazione Istituto Italiano di Tecnología, via Morego 30, 16163 Genoa, Italy*Correspondence: martina.pannuzzo@iit.it

Advances in computer science and the development of new methods for large-scale Molecular Dynamics (MD) simulations are offering new insights that help rational design of nanoscale drug carriers for site-specific targeting. This work uses MD for improved understanding of the dynamic interplay between polymeric drug carriers, drugs, biological membrane and proteins. First, PLGA and PEO/PEG mixtures are simulated through MD with the aim of elucidating the mechanisms regulating nanoparticle deformability, a parameter which is known to modulate the biological performance of intravenously injected nanocarriers. Atomistic simulations are used complementarily with differential scanning calorimetry to investigate the miscibility of the two species as function of relative molecular weight and concentration. Then, the mechanical strength of an immiscible and a partially miscible polymer blend is estimated. Once the carrier has been structurally characterized, enhanced sampling techniques are employed to estimate the free energy profile of the translocation of the drug from the polymer carrier into the aqueous solvent. Indeed, drug release kinetics is modulated by physico-chemical interactions between the drug and the polymer matrix. Finally, with the purpose of overcoming limitations in size and time scales, a multiscale approach (all-atom and coarse-grained resolutions) is employed to integrate in vivo and in vitro experiments to elucidate the complex interactions at the particle-membrane interface or adsorbed proteins-particle interface.

Together, this integrated multi-parametric simulation-biophysical approach has set the rationale for accelerated polymeric drug carrier engineering for therapeutic interventions.

### 2.2. Session B: Can DDS Meet Clinical Needs in Ophtalmology?

#### 2.2.1. Drug Delivery Strategies for Ophthalmic Diseases

RuscianoDarioResearch Director of Sooft Italia SpA, 63833 Montegiorgio (FM), Italy; dario.rusciano@sooft.it

Several barriers protect the eye from external insults, and therefore make the eye hardly accessible to drug administration. The epithelial barrier of cornea and conjunctiva shields the eye from the outside, and the blood retinal barrier shields it from inside intrusions through the vascular endothelium. Moreover, the continuous blinking of the eye reduces the permanence time of any eye drop given to the eye surface, and therefore its penetration chances. Therefore, specific drug delivery strategies and/or specific molecular drug design must be used to improve the pharmacokinetics of ophthalmic drugs.

Nanotechnologies are greatly helping drug delivery through the epithelial barrier, and the use of nano-liposomes, lipid nanoparticles or nanomicelles has resulted in significant improvements of the retention time and penetration efficiency of different classes of drugs given as eye drops on the ocular surface. Not only drug delivery to the anterior segment of the eye, but also to the posterior segment and the retina have been ameliorated by these techniques. Interesting results have also been obtained by chemical derivatization of known antioxidant molecules, to help either their incorporation into liposomes, or their direct penetration through the epithelium.

Several examples taken from our personal experience and some from the scientific literature will be shown to illustrate the different possible strategies to achieve more efficient therapeutic effects in eye pathologies.

#### 2.2.2. The Challenges and Opportunities of Drug Delivery to the Eye

BrownMarc B.CSO and Co-Founder MedPharm Ltd., Guilford GU27AB, UK; marc.brown@medpharm.co.uk

The prevention and treatment of diseases of the eye represents a US $23 billion (£18.2 billion) annual market globally. The major impact eye health and vision make on quality of life means that this is a rewarding area for development of any drug products that meet a medical need. Modern lifestyles and an ageing population are also bringing the need for solutions to ocular diseases, such as dry eye disease (DED), to the attention of the ophthalmology community. The eye is a distinct organ with the possibility of direct access and thus ocular drug delivery presents both unique challenges and opportunities.

The current and future status of topical, intra-ocular and peri-ocular drug delivery has been reviewed in this presentation along with an explanation of in vitro and ex vivo performance testing models that are being developed to reduce the risk of ocular drug product failure in the clinic.

#### 2.2.3. Topical Drug Delivery Systems to Tackle Ocular Diseases

Alvarez-LorenzoCarmenDepartamento de Farmacología, Farmacia y Tecnología Farmacéutica, I+D Farma (GI-1645), Facultad de Farmacia and Health Research Institute of Santiago de Compostela (IDIS), Universidade de Santiago de Compostela, 15782 Santiago de Compostela, Spain; carmen.alvarez.lorenzo@usc.es

Delivery of drugs to the ocular structures must overcome numerous anatomical barriers and eye defense mechanisms. The efficacy of systemic administration is limited by the blood-ocular barriers. Periocular and intra-ocular injections allow treating diverse ocular pathologies, but risks associated with their administration prompt the development of more efficient topical formulations. Indeed, topical formulations are comfortable and safe, but the drug ocular bioavailability is limited to 1–10% of the dose. Low cornea permeability, short residence time, rapid tear fluid turnover, and efflux pumps negatively affect drug absorption. To overcome these hurdles, two different strategies are under investigation: nanocarriers and contact lenses.

Drug nanocarriers have advantageous properties for both anterior and posterior segments treatments. In this regard, polymeric nanomicelles are particularly appealing [10]. They are spontaneously formed, encapsulate hydrophobic drugs, withstand the dilution in the lachrymal fluid and favor drug partition toward the corneal epithelium. Moreover, unimers may inhibit efflux pumps. The nanomicelles can penetrate through different pathways or undergo in situ gelling transitions favoring ocular permanence. Moreover, drug-loaded polymeric micelles may reach the posterior segment of the eye through the conjunctival–scleral route. Relevant examples of nanomicelles for lipoic acid, acyclovir [11], or cyclosporine ocular delivery are discussed, paying attention to the pre-clinical tests suitable for predicting in vivo performance.

**Figure 3:**
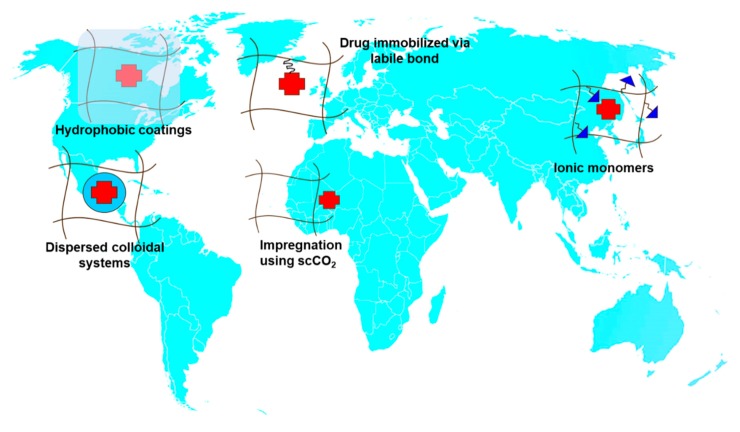
Worldwide strategies to produce drug-eluting contact lenses.

Contact lenses (CLs) are the devices that can stay on the eye for more time with good patient compliance. The therapeutic benefits that CLs can offer as drug release platforms are enormous since controlled concentrations of drug on the cornea surface may greatly enhance ocular bioavailability while minimizing loss of drug by premature clearance or by unwanted systemic absorption, resulting in improved efficacy with minimized collateral effects [12]. Approaches to endow CLs with capability to host drugs and control their release are discussed paying special attention to bioinspired strategies [13] (Figure 3).

**Acknowledgments:** This work was supported by MINECO [SAF2017-83118-R], Agencia Estatal de Investigación (AEI) Spain, Xunta de Galicia [ED431C 2016/008] and FEDER.

### 2.3. Session C: Relevance and State of the Art of Clinical Studies in Drug Release and Targeting

#### 2.3.1. The Regenerative Potential of Stem Cell-Derived Extracellular Vesicles: The Challenge for Shifting from Pre-clinical to Clinical Applications

CamussiGiovanniDipartimento di Scienze Mediche, Università di Torino, 10126 Torino, Italy; giovanni.camussi@unito.it

**Figure 4:**
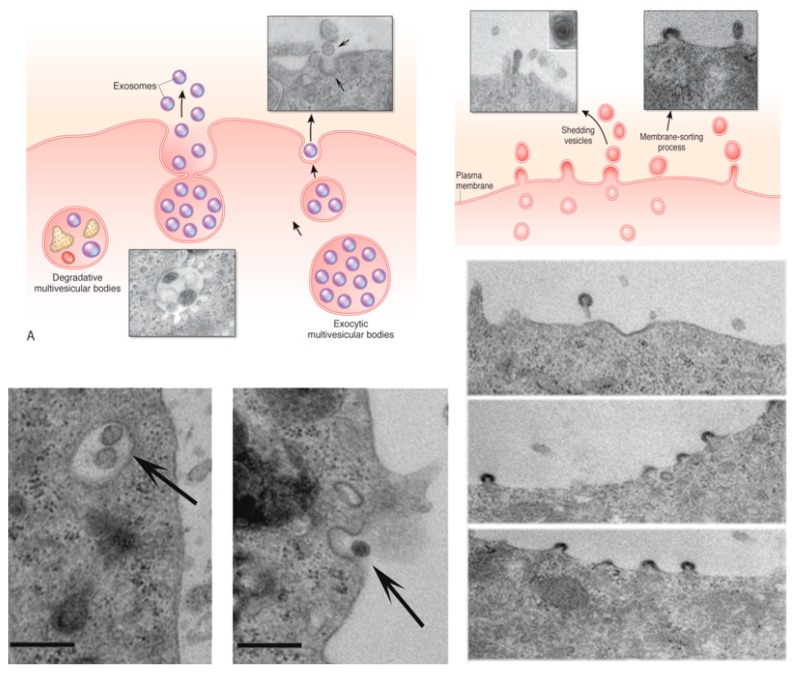
EVs from stem /progenitor cells as a paracrine/endocrine mechanism.

Extracellular vesicles (EV) are membrane vesicles comprising exosomes derived from the multivesicular bodies and ectosomes released by budding of plasma membrane (Figure 4). EV composition is complex as they carry the recipient cell biologically active lipids, proteins and nucleic acids. The transfer of molecular information may modify the phenotype and the function of the recipient cell in both physiological and pathological conditions. The biological action of EVs depends not only on their molecular content but also on pathways activated in the recipient cells.

Stem cells as many other cell types may release EVs with autocrine and paracrine activities. Many pre-clinical studies have shown that stem-cell-derived EVs may mimic the beneficial effect of the cell of origin suggesting a possible therapeutic exploitation. However, several problems should be solved before entering the feasibility of a clinical application. The first critical point is the large-scale production of EVs in GMP conditions. The second is the definition of EV characterization and classification of an EV-based therapy. For what concerns characterization, we should consider that EVs are a heterogeneous population defined by its size in the nano range. EVs also express surface markers of the cell of origin as well as some exosome specific markers that may serve as a test of identity.

Moreover, it is critical to define formulation, storage condition, stability and techniques that may characterize the consistency and purity of EV preparations, and more importantly, to have validated tests of potency specific for different clinical applications.

#### 2.3.2. Clinical Testing in Children: The Case of Givinostat in Duchenne Muscular Dystrophy

BetticaPaoloItalfarmaco Group, 20126 Milan, Italy; p.bettica@italfarmaco.com

Italfarmaco is developing Givinostat for the treatment of Duchenne Muscular Dystrophy (DMD), a rare degenerative, X-linked recessive genetic disorder with estimated incidence of 1 in 5000 live births, caused by mutations in the dystrophin gene.

In DMD, mutations in the dystrophin gene disrupt the open-reading frame, resulting in an absence of functional dystrophin, a critically important part of the protein complex that connects the cytoskeletal actin of a muscle fiber to the extracellular matrix. Lack of dystrophin causes repetitive muscle damage. In a normal muscle, the physiological repair process removes the damaged muscle fibers and reconstitutes normal muscle fibers. In DMD, on the other hand, the repair process leads to a combination of new muscle fibers, fatty replacement and fibrosis, with the two latter components progressively prevailing as a process called fibroadipose degeneration. This detrimental outcome typically compromises muscle function, alters the tissue environment, and probably limits the potential effectiveness of regenerative approaches.

As described by Peverelli et al., fibrosis is already significant at one year of age in DMD patients constituting approximately 16% of a muscle biopsy (3% in age matched controls) and remains relatively constant until 7 years of age. At this age, there is an important increase in the proportion of fibrosis that reaches approximately 30% of muscle biopsy between 7 and 10 years of age. Similarly, MRI studies have shown that fat infiltration is already present at 5 years of age and constantly increases.

DMD is characterized by progressive symmetrical muscular weakness that affects proximal muscles more than distal muscles, often accompanied by calf muscle pseudo hypertrophy. Significant motor deficits may be present during the first year of life, but diagnosis is usually made between the ages of 3 to 5 years when the affected patients begin to show waddling gait, toe walking, and difficulty climbing stairs. Over time, ambulation becomes increasingly abnormal, and by 8 years of age, most patients lose the ability to rise from the floor and climb stairs, and often fall while walking. By 12 to 14 years of age, most lose the ability to walk and the heart and respiratory muscles also are affected. Upper limb, cardiac, and diaphragmatic muscles progressively weaken during adolescence. After 18 years all patients are affected by cardiomyopathy. Only a few survive beyond the third decade; most patients die because of respiratory complications and heart failure due to cardiomyopathy. There are still no curative treatments for such a debilitating disease and the current management of the disease is based on prevention and management of complications.

Corticosteroids (e.g., prednisone or deflazacort) have been demonstrated to slow the rate of muscle weakness when initiated in ambulatory boys, however, the use of corticosteroids is limited by all the potential side effects, including weight gain, cataracts, osteopenia, and avascular necrosis. Recently, the European Medicines Agency has granted a conditional approval to Ataluren, which targets nonsense mutations as the cause of DMD, which is relevant for only 13% of DMD population, or approximately 2000 patients in the United States (US) and 2500 patients in the European Union (EU). In addition, the FDA has granted an accelerated approval to Eteplirsen, which targets DMD gene mutations skippable with exon 51 skipping, which are relevant for only 13% of DMD population, or approximately 2000 patients in the United States (US) and 2500 patients in the European Union (EU). In light of the above considerations, it becomes evident that an unmet therapeutic need exists for the treatment of this disabling and fatal condition.

Givinostat is an HDAC inhibitor which has been shown both in a pre-clinical model and in a clinical study to counteract the histological changes induced by the lack of dystrophin in muscles. Based on these results, Italfarmaco has initiated a phase III registration study (the Epidys study) which is currently ongoing.

#### 2.3.3. Unique Features of Pediatric Drug Delivery

ColomboGiuseppeItalfarmaco SpA, 20126 Milan, Italy; giuseppe.colombo@italfarmaco.com

In the past, many authorized medicines were not studied adequately or authorized in children. Children represent a vulnerable population group with developmental, physiological and psychological differences from adults. They are not merely “small adults”.

Age- and development-related research, and the availability of suitable medicinal products, is consequently particularly important.

The presentation outlines the unique feature of pediatric drug delivery. A medicine designed for use in Pediatric Patients must consider in fact the following:

(1) Patient population variability (age development)
(2) The need for dose flexibility
(3) Excipient tolerability
(4) Easy and safe administration
(5) Patient and parent compliance (dosage form child can take/caregiver can administer).

Eventually the case of the development of a Givinostat oral liquid formulation for use in Duchenne Muscular Dystrophy (DMD) is discussed. DMD is a rare genetic disease that causes the muscles in the body to become weak and damaged over time and is eventually fatal. The symptom of muscle weakness usually begins around the age of four in boys and worsens quickly. Most are unable to walk by the age of 12. By the early teens, the heart and respiratory muscles also are affected. DMD is caused by mutations in the dystrophin gene leading to dystrophin deficiency, muscle fiber degeneration and progressive replacement of muscle with fibrotic tissue.

Givinostat, a histone deacetylase (HDAC) inhibitor, was shown to significantly reduce fibrosis and promote compensatory muscle regeneration in mdx mice. This presentation highlights the main development efforts leading to the obtainment of an acceptable oral liquid formulation for clinical development. Particularly it will be focused on reasons leading to the selection of the oral liquid formulation as “dosage form of choice”, such as appropriate volume of administration (≥0.5 mL and ≤10 mL), wide dosage flexibility (that largely covers the anticipated doses for the clinical study) and an acceptable chemical stability at refrigerated conditions.

#### 2.3.4. Gene Therapy from Pre-clinical Development to Clinical Application

AlhakamyNabil AbdulhafizDepartment of Pharmaceuticals, Faculty of Pharmacy, King Abdulaziz University, Jeddah 21589, Saudi Arabia; nalhakamy@kau.edu.sa

Gene therapies rapidly become a key component of the therapeutic strategies for a wide range of inherited and acquired human diseases after almost three decades of promise tempered by setbacks. Gene therapies for inherited diseases, hemophilia, neurodegenerative diseases, and cancers have lately advanced to approved drug status in China, Russia, the European Union, and the United States of America, Canada, or are expected to obtain approval soon. Recent gene therapy clinical trials have demonstrated notable therapeutic advantages and an outstanding record of safety. To date, more than 3000 directly related to gene therapy clinical trials have been finished, are in progress or approved throughout the world. Our database collects worldwide data on clinical trials of gene therapy from global trial databases and formal agency sources (e.g., International Clinical Trials Registry Platform (ICTRP), NIH ClinicalTrials.gov, EU Clinical Trials Register (EU-CTR), and others).

**Figure 5:**
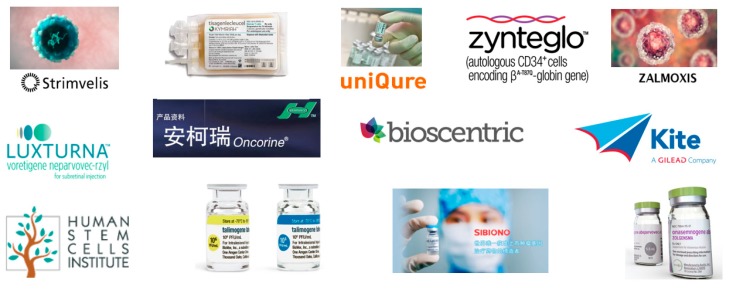
Some of the gene therapy-based products that have reached the clinical market.

Here we present our evaluation of the directly related to the gene therapy clinical trials that have been or are being conducted globally to the best of our understanding. Moreover, we will discuss milestones in the basic process and types of gene therapy, the vectors for gene delivery, and the clinical trials regulatory affairs of gene therapy. We will also discuss gene therapy products (approved and clinical trials) and gene therapy challenges (Figure 5). Additionally, we will cover emerging technologies for genome editing that would further progress the scope and efficiency of approaches to gene therapy.

#### 2.3.5. Prodrugs for Overcoming Pre-Clinical and Clinical Issues

RautioJarkkoSchool of Pharmacy, University of Eastern Finland, 70210 Kuopio, Finland; jarkko.rautio@uef.fi

The current interest in prodrugs is evident. In the past 10 years, the FDA has approved over 30 prodrugs, and approximately 10% of all marketed drugs worldwide can be considered prodrugs. Prodrug strategies are versatile and powerful tools to improve the problematic characteristics of molecules. Those have traditionally been embarked to address ADME properties and risks of marketed drugs or as a tool in late-stage problem solving for drug candidates in development phases.

However, prodrug design is now being integrated into early drug discovery. Admittedly, embarking a prodrug strategy can certainly present its own challenges, but depending on the chemical nature of the parent drug and the therapeutic target, many times the prodrug design can represents a comparable smaller challenge than the alternative of searching for a new therapeutically active molecule that also inherently possesses the desired ADMET properties.

This presentation discusses various prodrug strategies to overcome pre-clinical and clinical issues.

### 2.4. Session D: Clinical Trials for Health Products: The Impact of UE Regulations

#### 2.4.1. Clinical Trials for Non-Pharmaceutical Products: The State of the Art

DragoValentinaSCF, 95125 Catania, Italy; valentinadrago@essecieffe.it

The not-pharmaceutical market is facing an exponential growth, in terms of usage and market expenditure. It has been estimated that the Italian market of food supplements has grown by 4% from 2018 to 2019, with volume sales of around €3.3 billion [14].

This means that there is a growing number of people relying on these products, based on their undoubted value, as food supplements do in circumstances of real needs to supplement the normal diet, even if sometimes they are wrongly seen as an “alternative” of medicinal products, often leading to an abuse or misuse of them.

**Figure 6:**
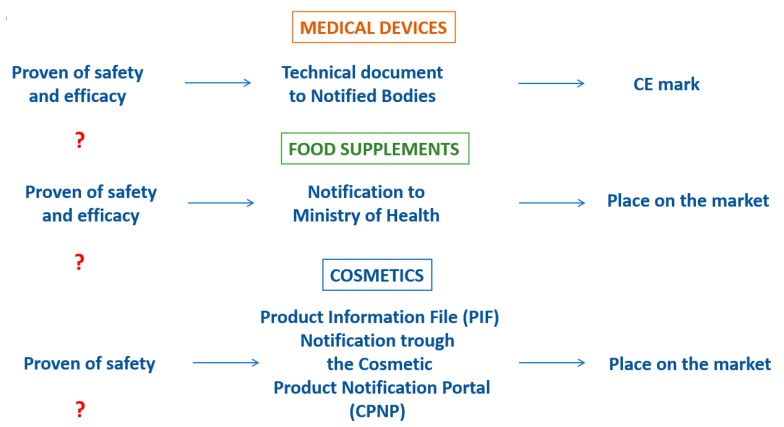
Clinical trials for not-pharmaceutical products: the state of the art (source: www.brainandlife.org).

There is a strong attention by the Health Authorities on the monitoring of their usage and safety issues that can be rise during the post-marketing phase. What about the pre-marketing phase? In contrast with the pharmaceutical products, clinical studies are not required for the marketing authorization about the food supplements, along with medical devices and cosmetics (Figure 6).

So, how a not-pharmaceutical product is considered safe and effective?

Even if the Directives and Regulations that governed these products (Directive 2002/46/CE for food supplements, Directive 93/42/CE and Regulation 745/17 for medical devices and the Regulation 1223/09 for cosmetics) defined the criteria, requirements and specific limitations for the manufactures, these does not refer to the needs of a proven of safety and efficacy through the conduction of clinical trials.

Despite this, in recent years there are a growing number of clinical trials about the not-pharmaceutical products, even though they are not required for the marketing authorization, except for such circumstances about the medical devices as required by the new Regulation 745/2017.

According to the Italian scenario, the conduction of clinical trials on food supplements and medical devices is subordinated to a notification to the Ministry of Health, which does not correspond to the clinical trial pre-evaluation phase.

Facing a growing of the market of the not-pharmaceutical products, the conduction of clinical studies should be enforced and should be standardized in terms of quality, as the GCP application already do for the pharmaceutical products. These studies are able to establish the safety and efficacy profile of these products ex-ante, and not just for the products per-se but also for the evaluation of the possible interaction can emerged from the combination of the pool of substances within the products, and between the not-pharmaceutical and pharmaceutical products co-administration.

#### 2.4.2. Drug Delivery Systems as Combination Products in Medical Device Development 

LeonardiAntonioLocate Bio Limited, MediCity, Thane Rd, Beeston, Nottingham NG90 6BH, UK; aleonardi@locatebio.com

**Figure 7:**
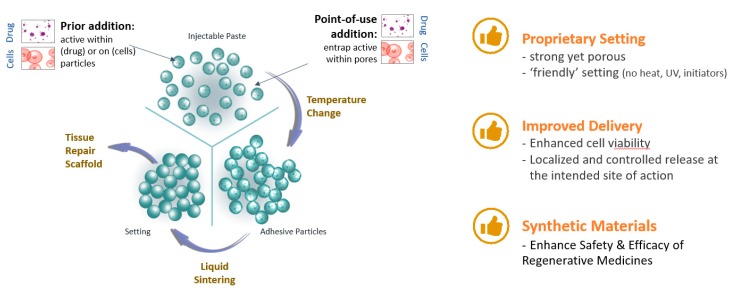
Mode of action of TAOS^TM^ (Targeted Orchestrated Signaling) matrix.

Locate Bio ltd. has developed a new class of injectable and biodegradable material and is using this technology to develop combination products, initially for use in orthopedic applications. This material is called TAOS™ (TArgeted Orchestrated Signaling). TAOS™ converts from a granular suspension to a highly porous solid within minutes under conditions that are benign to human tissue (Figure 7). It forms a regenerative matrix that is >70% porous with pore sizes optimized to host tissue growth. The system is activated by addition of an aqueous liquid (e.g., saline, biological fluids) and the mechanical properties and degradation profile of the final matrix can be tuned to the requirements of the indication. Furthermore, this matrix can deliver active agents such as APIs, proteins and cells, making it a versatile platform for regenerative medicine and for treating diseases/conditions that require a localized and controlled release at the intended site of action.

The full development process will be described from conceptualization to final introduction to the market, which companies adopt to develop either medical devices (MD) and combination products (drug/device and biologic/device), such as TAOS™. Attention will be given to the in vitro and in vivo trials chosen to prove safety and efficacy, as well as the design/prototype validation and verification process developed to meet the regulatory requirements. Key considerations around the decision-making process for pre-clinical testing (purpose, objective, and endpoints of the studies) will be discussed since this is a fundamental step for the development of MD and combination products. In particular, ISO 10993 biocompatibility testing; appropriate animal models; type and number of test/s and control/s and relevant in-life and ex vivo analysis will be examined.

The concept of risk management will also be introduced including the adoption of ISO 14971 as an integral part of the product development lifecycle that aims to reduce or mitigate the chances of product failure and avoid any possible hazards associated with the device.

**Acknowledgments:** This work was supported by direct investments from Mercia Technologies PLC and by three grants from Innovate UK: (1) A Targeted and Orchestrated Signaling Matrix for Clinically Challenging Defects. Project No: TP101619; (2) Pre-clinical Proof of Concept for a Cell Therapy Delivery Matrix. Project No.: 132217; (3) CellFuse: A Regenerative Medicine Product for Enhanced Spinal Fusion in Diabetic Patients. Project No.: 103861.

#### 2.4.3. Novel Delivery Approaches for Nutraceuticals

MasonVeraNutraceutical Research and Innovation Technology Department, Labomar Research, 31036 Istrana (TV), Italy; vera.mason@labomar.com

In recent years, nutraceutical products have gained increasing attention due to their role in preventing or treating nutritional disorders and pathological conditions. The constituents of nutraceutical formulations include vitamins, plant-derived active ingredients, coenzymes, fatty acids and micronutrients with proven beneficial effects on human health. However, the efficacy of such formulations depends on the effective absorption of the active ingredient by the gastro-intestinal tract and on its metabolization. In fact, many of nutraceutical ingredients exhibit poor or incomplete bioavailability because biophysical and biochemical mechanisms limit enteric absorption.

The development of adequate delivery systems can improve the bioavailability of nutraceutical compounds to optimize the efficacy of the product. According to substrate features and to the desired pharmaceutical form of the finished product, different approaches can be developed. Nanoemulsion delivery system and adsorbed nanoemulsion system (Labomar Research, Istrana, TV, Italy) promote bioavailability of small lipophilic active ingredients through the sublingual route. These technologies entrap oily compounds in stable liquid and powder systems and increase water dispersion of the substrates assuring their absorption through the tongue mucosa.

Enterosoma technology (Labomar Research, Istrana, TV, Italy) acts on physiological barriers and modulates enteric absorption. The gastroresistant tablet reaches the small intestine and the interaction with P-Glycoprotein (Pg-P) pump and tight junctions favors active ingredient internalization.

Lipomatrix delivery system (Labomar Research, Istrana, TV, Italy) enhances the absorption of oils and lipophilic compounds through a lipid-based matrix. This innovative delivery platform, with a unique pool of excipient, embeds the active ingredients into a gastric-refractory system and once reached the small intestine promotes their emulsification.

**Acknowledgments:** The author would like to thank LABOMAR RESEARCH SPA the main source of funding for this work and owner of the patented technologies.

## 3. Poster Presentations

### 3.1. Hyaluronan-Based Nanogels as Trojan Horse. Chasing Intracellular S. aureus in Human Keratinocytes

MontanariElita^1^OatesAngela^2^MeoChiara Di^1^CovielloTommasina^1^ManciniPatrizia^3^MoscaLuciana^4^MatricardiPietro^1^*1Department of Drug Chemistry and Technologies, University of Rome, p.le A. Moro, 5, 00186 Roma, Italy2School of Healthcare, Faculty of Medicine and Health, University of Leeds, 13 Beech Grove Terrace, Woodhouse, Leeds LS2 9DA, UK3Department of Experimental Medicine, University of Rome, p.le A. Moro, 5, 00186, Roma, Italy4Department of Biochemical Sciences, University of Rome, p.le A. Moro, 5, 00186, Roma, Italy*Correspondence: pietro.matricardi@uniroma1.it

Several pathogens (e.g., *S. aureus*) are able to invade and persist in a range of cell types (e.g., keratinocytes); this adaptation may offer protection from the immune response and be a factor in treatment failure due to the inability of the antibiotics to target intracellular microorganisms. The incorporation of antimicrobials into hyaluronan-cholesterol nanogels (NHs) represents a novel paradigm in the delivery of therapeutics against intracellular pathogens. Sterile and antibiotics-loaded NHs (gentamicin, GM or levofloxacin, LVF) were achieved using an autoclave. NHs, GM/NHs or LVF/NHs were characterized in terms of shape, size and ζ-pot. MIC of GM/NHs or LVF/NHs was evaluated, first, against planktonic *S. aureus*, secondly, against the intracellular pathogen. The binding/uptake kinetics and the intracellular fate of NHs in HaCaT were studied.

**Figure 8:**
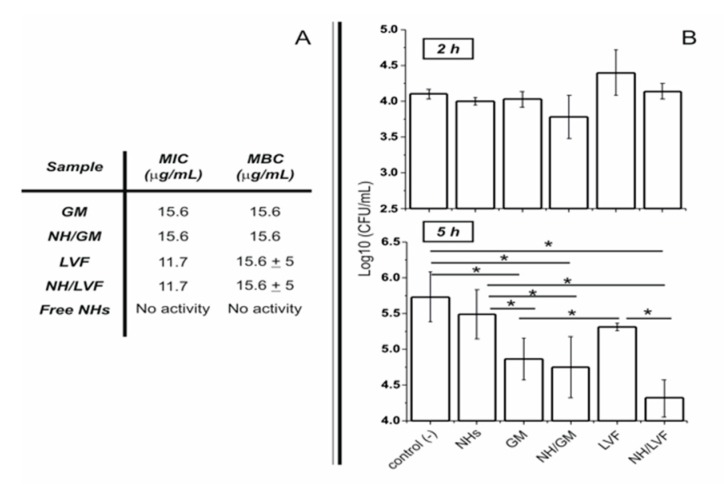
MIC of GM/NHs or LVF/NHs against planktonic (**A**) or intracellular (**B**) *S. aureus* in HaCaT.

Free or antibiotic-loaded NHs were formulated with a sterile autoclaving cycle. Loaded NHs displayed the same MIC or MBC as free LVF or GM against planktonic *S. aureus* (Figure 8A). Intracellularly, the antibacterial activity of LVF was highly enhanced by NHs (Figure 8B). NHs quickly entered HaCaT and co-localized with lysosomes. *S. aureus* can survive and accumulate in lysosomes. Free LVF predominantly accumulates in the cytosol. As NHs enhanced the intracellular activity of LVF, these results strongly suggest NHs may change the intracellular fate of LVF, targeting to intracellular *S. aureus*. Indeed GM, which predominantly accumulates in lysosomes, displayed a significant intracellular activity without the employment of NHs. This research demonstrates that sub-cellular targeting may be essential for defeating intracellular microorganisms.

**Acknowledgments:** The authors acknowledge financial support from Sapienza University of Rome (“Finanziamenti di Ateneo per la Ricerca Scientifica—RP116154C2EF9AC8” and “Progetto di Ricerca RM11715C1743EE89”).

### 3.2. In Vitro and in Vivo Evaluation of Dexamethasone Loaded Oligocationic Liposomes in Retinal Diseases

Al-AminMd.^1^BalassoAnna^1^MarryStephen^2^ChenMei^2^TangMiao^2^UrttiArto^3^XuHeping^2^MastrottoFrancesca^1^CalicetiPaolo^1^SalmasoStefano^1^*1Department of Pharmaceutical and Pharmacological Sciences, University of Padua, Via F. Marzolo 5, 35131 Padua, Italy2Dentistry and Biomedical Sciences, Queen’s University Belfast, School of Medicine, 97 Lisburn Road, Belfast, BT97BL3Division of Pharmaceutical Biosciences, University of Helsinki, Viikinkaari 5 E, 00014, 00100 Helsinki Finland*Correspondence: stefano.salmaso@unipd.it

Retina is an integral part of the eye responsible for vision and various diseases are associated with retinal degeneration [15]. Unique anatomy of the eye poses challenges to efficient delivery of therapeutics to the retina [16]. Surface decorated liposomes represent a valid delivery strategy to improve residence time of drugs in the vitreous, thus reducing administration frequency, and effective interaction with retinal barrier to facilitate intracellular access [17]. In this study, we aimed at modulating the surface properties of liposomes with a combination of mPEG_2 kDa_-DSPE and a newly synthesized oligocationic non-peptidic non-linear cell penetration enhancer (CPE) to control both their diffusivity in the vitreous and intracellular access. The nano platform has been used to deliver the anti-inflammatory agent dexamethasone by intravitreal administration.

Dexamethasone loaded liposomes were prepared by remote loading approach using calcium acetate gradient. A variety of formulation parameters were investigated to assess their effect on the loading efficiency and capacity, and colloidal features. Dexamethasone loaded liposomes were decorated with 5 mol % CPE and 5 mol % mPEG2 kDa-DSPE. Cryo-EM analysis has been performed in various liposomal formulations. In vitro release and stability studies have been carried out in buffer at pH 7.4 and 37 °C. In vitro cyto-toxicity and anti-inflammatory activity of liposomes were tested in ARPE19 cell line. In vivo efficacy of the liposomes was evaluated by intra-vitreal injection of the formulations in a C57BL/6 mouse model after laser induced choroidal neo-vascularization in retina.

Dexamethasone hemisuccinate loaded liposomes were successfully fabricated with a size of ~170 nm and low PDI (<0.1). The CPE decorated liposomes showed a positive zeta potential (+13 mV), while CPE/PEG-coated liposomes displayed a slightly positive zeta potential of +3.7 mV because of PEG shielding of the CPE. Cryo-EM analysis demonstrated the presence of dexamethasone-calcium rod shape matrix in the aqueous phase of the liposomes similarly to Doxyl. In vitro release studies demonstrated a slow release of dexamethasone for 20 days. Each formulation was colloidally stable over 20 days indicated by no significant changes in size and P.D.I. The liposomal formulation was not toxic to the retinal cells and dose dependent decrease of pro-inflammatory cytokine (IL-6) was observed in LPS induced cell model. Ongoing in vivo test will assess the effect in reducing the neovascularization process by looking at the eye fundus and quantifying the vascularization area with respect to controls.

In conclusion, in this study dexamethasone loaded liposomes were generated and their in vitro biopharmaceutical profile has been investigated. The modulation of surface properties of liposomes by multifunctional components represents a relevant strategy to enhance their residence time in the vitreous and while promoting access to target cells for anti-inflammatory drug delivery. This surface decorated liposomal delivery system might be a promising approach to treat retinal diseases.

**Acknowledgments:** This project has received funding from the European Union’s Horizon 2020 research and innovation program under the Marie Skłodowska-Curie grant agreement N° 722717.

**Conflicts of Interest:** The authors declare no conflict of interest. The founding sponsors had no role in the design of the study; in the collection, analyses, or interpretation of data; in the writing of the manuscript, and in the decision to publish the results.

### 3.3. Pentamidine-Loaded Lipid and Polymer Nanocarriers as Tunable Anticancer Drug Delivery Systems

AndreanaIlaria*StellaBarbaraArpiccoSilviaDipartimento di Scienza e Tecnologia del Farmaco, Università degli Studi di Torino, Via P. Giuria 9, 10125 Torino, Italy*Correspondence: ilaria.andreana@unito.it

The class of diamines is widely known for the antimicrobial activity and, recently, some of them have been proposed in the treatment of different cancer types [18]. To efficiently deliver these molecules and reduce their side effects, lipid and polymer nanosystems have been proposed as useful delivery systems. Here we propose the formulation of pentamidine in liposomes and poly(lactide-*co*-glycolide) (PLGA) nanoparticles and the comparison of the physiochemical characteristics of the loaded nanocarriers.

Liposomes were formulated by thin lipid film hydration method followed by extrusion; pentamidine-loaded nanoparticles were prepared by the nanoprecipitation technique. Nanosystems were characterized concerning the size, zeta potential, physical stability, morphology (CryoTEM), pentamidine loading and drug release profile. The anticancer activity was evaluated on a human ovarian cancer cell line over 72 h.

Different formulations were compared to obtain non-toxic, biocompatible and biodegradable nanosystems for the optimal delivery of pentamidine. Results showed that the drug is efficiently loaded into liposomes with different counter ions (using transmembrane citrate- or sulfate-gradient) [19]. Concerning PLGA nanoparticles, ionic interactions between the drug and the polymer occurred and the formulation was characterized by high encapsulation efficiency [20]. The in vitro tests confirmed pentamidine anticancer activity; moreover, its release profiles depend on the drug form and the nanocarriers’ structure.

In conclusion, the nanocarriers proposed could be considered to be a platform for pentamidine delivery and they can increase the therapeutic application of the drug. 

**Acknowledgments:** This research was funded by Italian Ministry for University and Research (MIUR)—Università di Torino, “Fondi Ricerca Locale (ex-60%)”.

**Conflicts of Interest:** The authors declare no conflict of interest.

### 3.4. Efficient siRNA Delivery Using Carbosilane Dendrimers for Overcoming Cancer Drug Resistance

ArgenzianoMonica^1^*RossiDavide^1^AmbrosioLeanne^2^GraafInge AM de^2^BarreraGiuseppina^3^RamírezRafael Gómez^4^PizzimentiStefania^4^CavalliRoberta^1^1Department of Drug Science and Technology, University of Turin, Via Giuria 9, 10125 Torino, Italy2Department of Pharmacy, University of Groningen, Antonius Deusinglaan, 1.9713 AV Groningen, The Netherlands3Department of Clinical and Biological Sciences, University of Turin, c.so Raffaello 30, 10125 Torino, Italy4Department of Organic Chemistry and Inorganic Chemistry, University of Alcalà, Campus Universitário Alcalá de Henares, 28805 Madrid, Spain*Correspondence: monica.argenziano@unito.it

The transcription factor Nrf2 (NF-E2-related factor 2) is the master regulator of antioxidant and cytoprotective systems. Nrf2 activation appears beneficial for carcinogen detoxification in normal cells; however, its activation is critical for resistance to drugs in various tumors, including bladder cancers. For this reason, it has been postulated that Nrf2 could represent an interesting target to combat chemoresistance. Since a small number of Nrf2 inhibitors have been identified so far, the use of a specific small interfering RNA (siRNA) against this gene is an attractive possibility [21]. However, siRNAs are unstable in blood and have very poor ability to cross the lipophilic cell membranes. Considering these limitations, the use of nanocarriers have been studied to protect siRNA from degradation during systemic circulation, and transport siRNA to target cells avoiding nonspecific delivery [22]. Dendrimers are repeatedly hyperbranched polymer molecules. The well-defined size and structure, branching architecture, and high density of tailorable surface functional groups can provide significant advantages. In this context, several types of cationic dendrimers have been explored for gene therapy, being able to form electrostatic complexes with nucleic acids. In particular, carbosilane dendrimers are emerging as attractive non-viral vectors to deliver siRNA both in vitro and in vivo [23]. The aim of this work was the design, development and the characterization of carbosilane dendrimer (CDD) nanoformulations for the delivery of siRNA against Nrf2. The biological activity of siRNA loaded carbosilane dendrimers in reducing drug resistance and tumor growth in bladder cancer cell lines with a high level of Nrf2 was also evaluated.

Stable nanosuspensions of CDD with sizes of about 300 nm were obtained. CDD were able to load siRNA and to protect it from degradation. siNrf2-CDD down-regulated the target gene in T24 cells and sensitized cisplatin-resistant cell lines to CDDP treatment. In conclusion, siNrf2-CDDs might represent a promising tool to overcome chemoresistance in bladder cancer.

### 3.5. Nanoemulsions as Delivery Systems for Poly-Chemotherapy Aiming to Melanoma Treatment

DianzaniChiara^1^CavallerisGiulia^1^CangemiLuigi^1^SerpeLoredana^1^MartinaKatia^1^MiglioGianluca^1^MiolettiSilvia^2^DianzaniUmberto^3^ClementeNausicaa^3^GigliottiCasimiro Luca^3^OsellaSara^4^BoggioElena^3^BattagliaLuigi^1^*1Università degli Studi di Torino, Dipartimento di Scienza e Tecnologia del Farmaco, via Pietro Giuria 9, 10124 Torino, Italy2Università degli Studi di Torino, Dipartimento di Scienze Veterinarie, via Leonardo da Vinci 10, 10095 Grugliasco (TO), Italy3Università del Piemonte Orientale, Dipartimento di Scienze della Salute, via Solaroli 17, 28100 Novara, Italy4Ospedale San Giovanni Bosco, Piazza del Donatore di Sangue 3, 10154 Torino, Italy*Correspondence: luigi.battaglia@unito.it

IV stage melanoma is the most advanced and critical stage, and leads to metastases or relapses, in the case of previous surgical removal. Despite the available current treatments being increased in recent years, current pharmacological therapies are only palliative care, which do not affect the final outcome, but whose primary end point is prolonging patient’s life. Therefore, the improvement of current chemotherapy is worthy of investigation.

In this experimental work a nanotechnology-based poly-chemotherapy aiming to treat IV stage melanoma is proposed and tested at pre-clinical level. Temozolomide, rapamycin and bevacizumab were co-loaded in injectable nanoemulsions for total parenteral nutrition (Intralipid^®^), owing to suitable devices, and preliminarily tested in vitro on human and mouse cell models and in vivo in B16-F10 melanoma mouse model.

The combination of drugs was efficiently loaded into the liquid lipid matrix of Intralipid^®^, including bevacizumab monoclonal antibody, leading to a fast internalization in tumor cells, as assessed through flow cytometry. An increased cytotoxicity towards melanoma cells, as well as an improved inhibition of tumor relapse, migration and angiogenesis was demonstrated in cell models for the Intralipid^®^ loaded drug combination. In preliminary in vivo studies, the proposed approach was able to reduce tumor growth significantly compared to controls. A relevant efficacy towards tumor angiogenesis and mitotic index was assessed, and immune response was probably involved.

In conclusion, in preliminary pre-clinical studies Intralipid^®^ proved to be a safe and versatile poly-chemotherapy delivery system for advanced melanoma treatment, by acting on multiple mechanisms, and allowing the perspective of a personalized nanomedicine.

**Acknowledgments:** The authors thank Italian Ministry of Education and University for funding (FFABR 2017, Ricerca Locale 2017-2018).

### 3.6. Mannose-Targeted Cationic Glycopolymers as New Tool for pDNA-Based Cancer Immunotherapy

BellatoFederica^1^FeolaSara^2^MaglioccaSalvatore^1^CalicetiPaolo^1^SalmasoStefano^1^CerulloVincenzo^2^MastrottoFrancesca^1^*1Department of Pharmaceutical and Pharmacological Sciences, University of Padua, Via F. Marzolo 5, 35131 Padua, Italy2Faculty of Pharmacy, University of Helsinki, Viikinkaari 5E, 00790 Helsinki, Finland*Correspondence: francesca.mastrotto@unipd.it

Tumor immunology is changing the landscape of modern anticancer therapy and the delivery of pDNA encoding Tumor Associated Antigens (TAAs) is emerging as a new strategy for anticancer vaccination. In this scenario, synthetic polymeric carriers have recently drawn increasing attention. In this work, Reversible Addition Fragmentation chain Transfer (RAFT) polymerization has been exploited for the synthesis of a small library of copolymers designed to deliver pDNA encoding TAAs to antigen presenting cells (APCs) and to trigger the immune response and memory against cancer. These polymers were designed such that Agm initiates nucleic acid electrostatic interactions and triggers formation of polyplexes that are stabilized by the presence in the outer layer of a mannosylated corona, thought to shield the polyplex charges in the core via steric hindrance mechanism, to confer stealth properties to the polyplex and, most importantly, to actively target mannose receptor (MR) expressed on APCs. Eventually, a hydrophobic butyl acrylate-based block (But) was introduced to provoke membrane disruption tuned to promote endosomal escape. Importantly, the system is also expected to provide nucleic acid protection against fast degradation, minimizing its interactions with nucleases.

Diblock Man58-b-Agm45 and a triblock Man62-b-Agm52-b-But32 cationic copolymers were generated by fast RAFT polymerization starting from D-Mannose acrylamide (Man) and Agmatine acrylamide (Agm), monomers and, for the triblock, performing the final chain extension with butyl acrylate (But). Polymers were found to fully complex model pDNA encoding for Enhanced Green Fluorescence Protein(pEGFP) at N/P ratios lower than 5 with the resulting glycopolyplexes (GPPs) being stable in the presence of physiological concentration of heparin. Furthermore, DLS and TEM characterization confirmed the co-existence of toroid-, rod- and spherical-shaped GPPs with a size distribution in the range of 100–1000 nm. In vitro flow cytometric studies revealed a remarkably high transfection of DC2.4 immortalized dendritic cells for Man58-b-Agm45/pEGFP and Man62-b-Agm52-b-But32/pEGFP, although the addition of the butyl-based block on the latter decreased the selectivity for MR-expressing cells, as shown by preliminary assays performed on model Chinese hamster Ovary cells (wild type CHO or mannose receptor expressing CHO-MR+). Finally, GPPs formulated with ovalbumin-encoding plasmid (pOVA) were found to efficiently stimulate the expression on DCs of the costimulatory clusters of differentiation CD86 and the presentation of the SIINFEKL ovalbumin antigenic epitope by MHC I molecule, with Man62-b-Agm52-b-But32/pOVA performing better than Man58-b-Agm45/pOVA. Despite that, therapeutic in vivo mice vaccination experiments highlighted Man58-b-Agm45/pOVA as the most promising candidate for cancer vaccine development, since it was able to control the tumor growth, and to efficiently induce the priming and the T cells specific activation against SIINFEKL antigenic peptide.

Future Perspectives: ongoing studies will investigate polymers suitability for mRNA complexation and delivery, thus enabling their exploitability for cell transfection and anticancer vaccination with a wide range of genetic therapeutics.

**Acknowledgments:** We acknowledge the University of Padua for financial support through the “STARS Starting Grants (STARS-StG)” (Grant No. MAST_STARS18_02; CUP G91I18001190005;) and the “Progetto di Ricerca di Dipartimento Junior—PRID-J” (Grant No MAST_SID2017_01; CUP C93C17002300005).

### 3.7. Development of Novel Super Stealth Immunoliposomes for Anticancer Drug Delivery

CanatoElena^1^*AlimontiA.^2^GuidoM.^3^GabbiaD.^1^MartinS. De^1^PasutG.^1^1Department of Pharmaceutical and Pharmacological Sciences, University of Padua, 35122 Padua, Italy2Institute of Oncology Research IOR, 6500 Bellinzona, Switzerland3Department of Medicine, Pathology and Cytopathology Unit, University of Padua, 35122 Padua, Italy*Correspondence: email: elena.canato@studenti.unipd.it

Advancement in the field of liposomes has resulted in the development of a great variety of nanocarriers, including long-circulating PEG-coated liposomes and targeted-liposomes. This work seeks to formulate new Super Stealth Immunoliposomes (SSIL), which should be both stable in the bloodstream and capable of reaching selectively the tumor site. PEG dendron molecules were conjugated to 2 or 4 molecules of distearoylphosphatidylethanolamine (DSPE) [24] to increase the hydrophobic interactions with the phospholipid bilayer. The conjugation of these PEG dendron-lipids derivatives to the Fab’ fragment of Trastuzumab, allowed to obtain liposomes selectively targeting the human epidermal growth factor receptor 2 (HER2), overexpressed on the surface of certain tumor cells.

PEG dendron-lipids derivatives were synthetized starting either from mPEG5kDa-NHS or Boc-NH-PEG5kDa-NHS by derivatization with β-glutamic acid and coupling to 2 or 4 molecules of DSPE. Boc-PEG5kDa-(DSPE)n derivatives, were further derivatized with N-(β-maleimidopropyloxy) succinimide ester (BMPS) to introduce a maleimide group. Trastuzumab was enzymatically digested with pepsin and reduced with cysteamine to yield the Fab’ fragment, which was immediately coupled to the maleimide moiety of PEG dendron-lipids derivatives. mPEG-lipid(s) derivatives and ligand-coupled PEG-lipid(s) derivatives were included into doxorubicin-loaded pre-formed liposomes by post-insertion. Liposomes were characterized by dynamic light scattering (DLS) and transmission electron microscopy (TEM). Drug leakage was quantified by measuring the fluorescence dequenching of doxorubicin (DXR) over the time. Preliminary cytotoxicity studies, pharmacokinetics and organ toxicity evaluation were performed to compare the in vitro and in vivo behavior of all the formulations.

Results showed that after 2 months of incubation at 4 °C and 25 °C all tested formulations were still stable and homogeneous. DXR was efficiently encapsulated (absence of leakage within 16 h). SSIL2 showed significantly prolonged elimination half-life (38.50 ± 8.53 vs. 12.49 ± 3.05 h of SL, *p* < 0.01) and reduced clearance rate (15.81 ± 3.90 vs. 48.04 ± 20.42 mL/h·kg of SL, *p* < 0.05). In HER-2 overexpressing cell lines (SK-BR3 and BT-474) SIL displayed a higher cytotoxic activity with respect to SL, thereby confirming the targeting effect of Trastuzumab. On the contrary, the IC50 of SSIL2 was higher than those of SL and SIL, probably because of PEG dendron molecules increasing the rigidity of the liposome bilayer thus affecting the uptake by the cells. Nevertheless in vivo SIL-treated rats showed numerous granulomatous lesions, sometimes associated with apoptotic bodies, whereas in SSIL2-treated animals only a few isolated granulomas could be observed. Neutrophil infiltration is indirectly confirmed by the increase of ROS concentration in the liver of rats treated with SIL (*p* < 0.001). Accordingly, CCL2 and IL-10, markers of macrophage infiltration, do not change in SIL or SSIL2 with respect to control rats, whereas CXCL2, a chemokine involved in the recruitment of neutrophils, was significantly higher in SIL-treated animals (*p* < 0.0001). Furthermore, the hepatic expression of IL-1β increased after treatment with immunoliposomes, although to a significantly lower extent in SSIL2-treated animals with respect to rats treated with SIL (*p* < 0.05). TNFα gene expression increased only in the livers of SIL-treated rats (*p* < 0.001), whereas was comparable to that of controls in SSIL2-treated rats.

In conclusion, SIL induce dramatic alterations in hepatic tissue, which is particularly rich of cells of the RES, probably due to their hepatic deposition. Conversely, SSIL2 caused only limited histological alterations in this organ and biochemical analyses confirm the lack of liver injury. Taken together, these data lead to the conclusion that SSIL2, besides their pharmacokinetic advantages, permit overcoming of the hepatic toxicity that can be associated with the administration of standard and stealth immunoliposomes, representing a smart strategy to improve the tolerability of cancer therapy.

### 3.8. Polysaccharide Hydrogels for the Stabilization and Controlled Release of Ionic Cargo

CasadidioCristina^1^CensiRoberta^1^*ScuriStefania^1^MayolLaura^2^BiondiMarco^2^RosaGiuseppe De^2^MartinoPiera Di^1^1School of Pharmacy, University of Camerino, Via S. Agostino 1, 62032 Camerino (MC), Italy2Department of Pharmacy, University of Naples Federico II, Via D. Montesano 49, 80138 Napoli, Italy*Correspondence: roberta.censi@unicam.it

Polysaccharides (PLS) are biodegradable and biocompatible and polymers, derived from renewable sources and showing great potential for several biomedical applications, such as the prevention and treatment of staphylococcal infections [25]. A strategy to fight these infections is the use of PLS-based delivery systems for the controlled release of antimicrobial drugs. PLS-based in situ-forming hydrogels are able to improve the efficacy of the loaded drug(s) while overcoming different drawbacks like: Systemic drug toxicity, high peak plasma concentrations and rapid drug degradation. In this work we investigated acidic PLS hydrogels as matrices for the stabilization and the release of vancomycin (VAN) [26,27].

Hyaluronic acid (HA), alginic acid (ALG), propylene glycol alginate (PGA) and xanthan gum (XA) were used as acidic PLS, while VAN was selected as drug. PLS-VAN solutions containing a fixed amount of polysaccharide (5 mg/mL) and PLS-VAN hydrogels at gel-point concentrations were formulated in phosphate buffer at pH 7.4. Chemical stability tests of VAN formulated into PLS-VAN solutions and hydrogels were performed for 22 days by HPLC-DAD-MS. Placebo hydrogels and drug-loaded systems were characterized by rheological analysis at 37 °C. VAN release studies were performed at pH 7.4 and 37 °C for 70 h. The antimicrobial activity of the complexes was tested against *Staphylococcus aureus* at 10^6^ CFU/mL after 24, 48 and 70 h via microdilution method.

**Figure 9:**
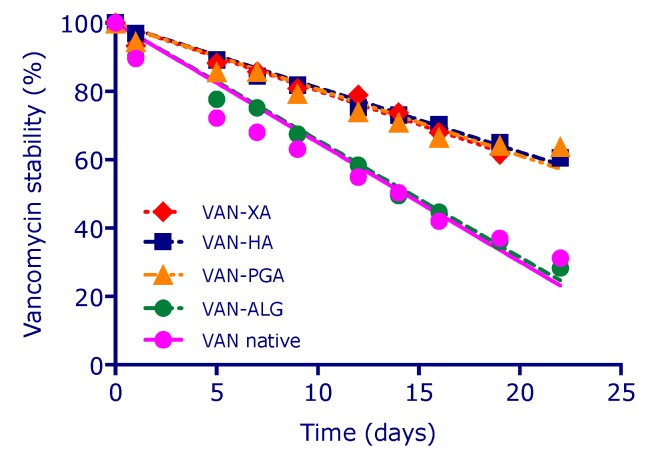
Residual concentration of native vancomycin within polysaccharidic hydrogels as a function of time.

VAN chemical stability tests showed that the glycopeptide antibiotic rapidly degrades via deamidation processes, when dissolved in phosphate buffer at pH 7.4, reaching a residual concentration of native VAN of 35% after 22 days. Conversely, when VAN is physically encapsulated into acidic polysaccharide networks at physiological conditions, it was found that the deamidation kinetics of the glycoprotein is decreased. In particular, XAN-VAN, PGA-VAN and HA-VAN hydrogels preserve VAN structure increasing with +25% its stability after 22 days compared with VAN solution (Figure 9). Moreover, results show that not all polymers were able to stabilize the drug to the same extent. VAN stabilization depends not only by physical encapsulation but also by ionic interaction between drug and polysaccharidic. In addition, the formation of ionic complexes affects rheological characteristics of the systems: the presence of the peptide confers to PGA and XAN gels an increase of both viscoelastic moduli while for HA formulation leads to a transition from an entangled solution to a gel-like behavior. Release tests revealed that at least 60% of VAN was released within 3 days for all formulations. Antimicrobials susceptibility tests show a significant bacteria reduction compared to VAN solution.

In conclusion, these designed formulations can preserve VAN structural stability enhancing its antimicrobial activity in physiological environment thanks to the physical encapsulation and self-assembling ionic complexes configuration. Furthermore, VAN-PLS gels could be potentially used as functional coating and as injectable hydrogels in the prevention and treatment of implant-associated or wound infections.

**Acknowledgments:** The authors acknowledge the E C for funding ISPIC—H2020-MSCA-ITN-2015 (Grant No. 675743); CHARMED—H2020-MSCA-RISE-2016 (Grant No. 734684); CANCER—H2020-MSCA-RISE-2017 (Grant No. 777682).

### 3.9. Solid Lipidic Nanoparticles Based on Naringenin and Linolenic Acid Viscosified with Biocompatible Polymers for The Transport and Release of Cyclosporine A

TrombinoSoniaServidioCamillaCurcioFedericaIemmaFrancescaCassanoRoberta*Department of Pharmacy and Health and Nutrition Sciences, University of Calabria, 87036 Arcavacata di Rende (CS), Italy*Correspondence: roberta.cassano@unical.it

**Figure 10:**
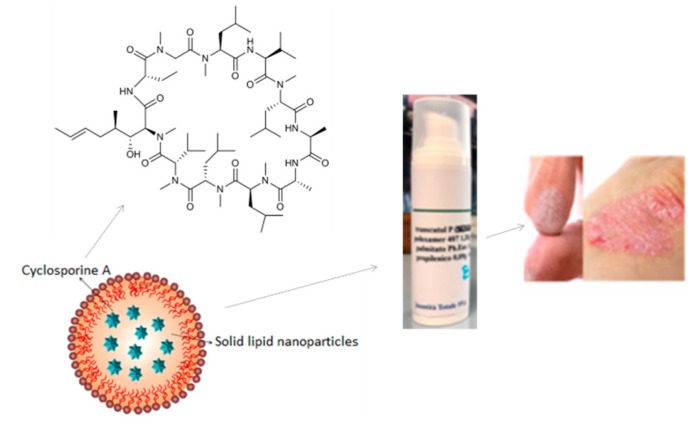
Schematic design of cyclosporin A-loaded SLN formulation.

This work aimed to design, prepare and study gels containing SLNs, based on an ester of the naringenin and linolenic acid, useful as cyclosporin A release system (Figure 10) [28,29,30].

**Figure 11:**
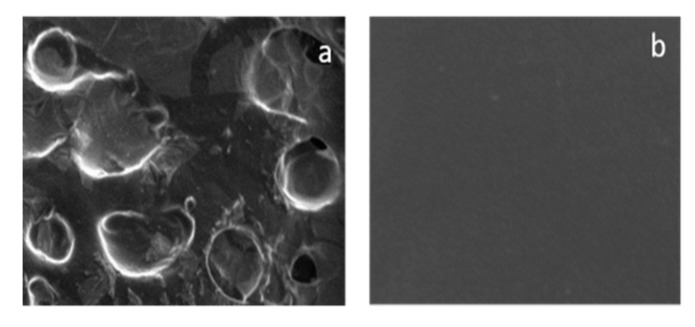
Photomicrography of empty SLN (Mag = 100 X) (**a**) and of gel based on HA and Poloxamer 407 containing empty SLN (Mag = 1.00 K X) (**b**).

**Figure 12:**
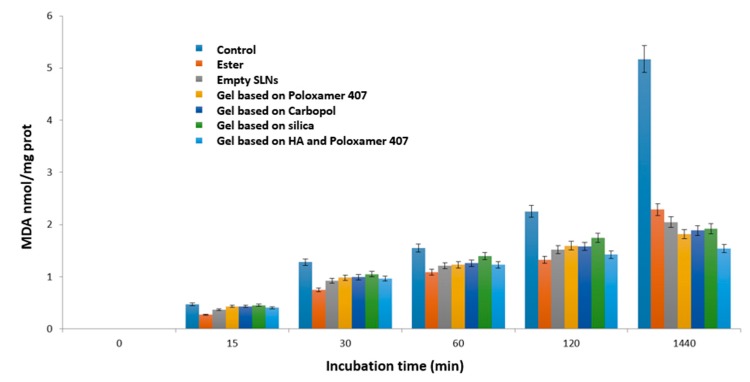
Inhibition of lipid peroxidation induced by *tert*-BOOH over 24 h.

The ester was characterized by FT-IR and 1H-NMR and the SLNs by Dynamic Light Scattering and Scanning Electronic Microscopy (Figure 11). Their capacity to inhibit the lipid peroxidation induced by a free radical generator, has been examined in rat liver microsomal membranes and compared to that of the free ester and various prepared gels, containing the empty lipid nanoparticles. All the materials were able to preserve the antioxidant capacity of the precursor (Figure 12) [31].

In particular, the major activity was exhibited by the free ester, the empty SLNs and the HA (hyaluronic acid)-based gel and Poloxamer 407 containing the empty SLNs [32]. This last result is due to the presence of the HA which also exerts an antioxidant action. However, even the other gels, despite being made up of non-antioxidant substances, show that they can preserve the microsomal membranes from lipid peroxidation due to the empty SLNs they contain.

**Figure 13:**
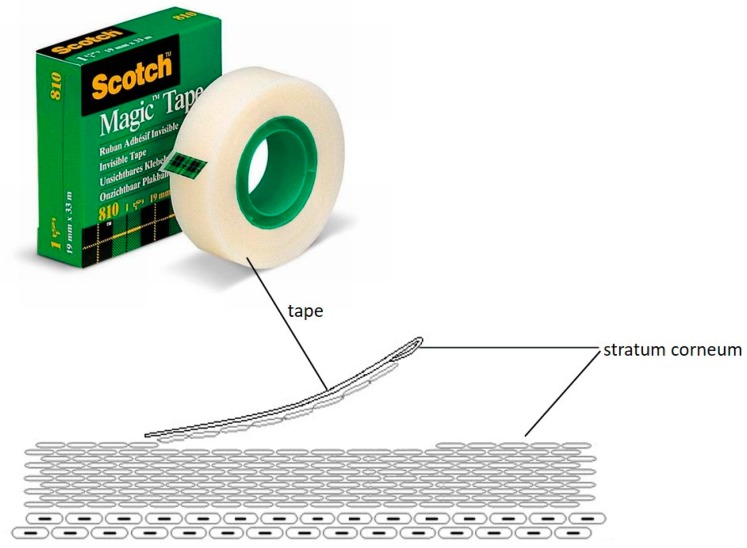
Tape stripping method.

Nanoparticles have been shown also to possess excellent encapsulation efficiency, stability and size suitable for topical administration. This hypothesis was supported by the results obtained with the transdermal release studies, performed using Franz cells, which revealed that in the case in which the SLN are incorporated in gels containing promoters of absorption, such as Poloxamer 407 and Carbopol, the gels release a maximum of 5% of the loaded drug in contrast to free SLNs and colloidal silica gels. To validate this hypothesis and evaluate the amount of cyclosporin A released in the stratum corneum and in the epidermis-dermis layer, the tape stripping method was used (Figure 13). The obtained data revealed that the amount of drug released by the colloidal silica-based gel was negligible in the stratum corneum (SC) and equal to 23% in the epidermis-dermis layer over 24 h. In contrast, the gel containing Poloxamer 407 at concentrations of 1.28% released about 79% of the drug over the 10 h in the SC and 15% in the epidermis-dermis layer. When the polymer changes, i.e., using Carbopol at 0.1%, the drug was more retained inside the gel than observed with the Poloxamer 407. In particular, the percentage of cyclosporin A released within 24 h was equal to 36% in the SC and 28% in the epidermis-dermis layer.

**Figure 14:**
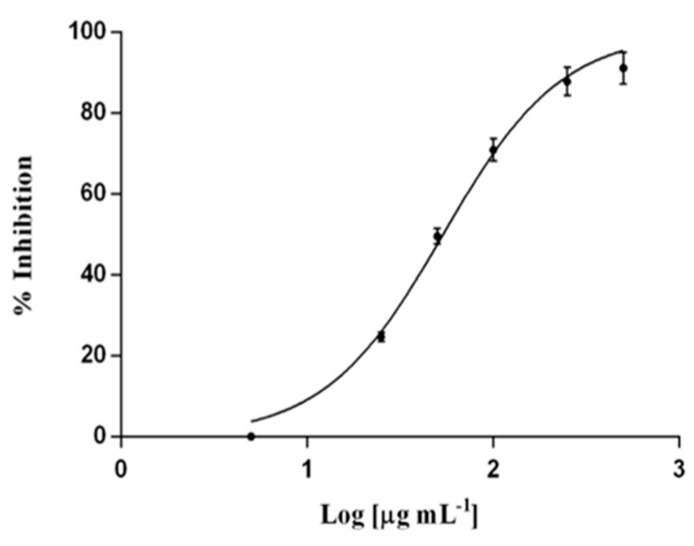
Inhibition of nitroxide production.

Furthermore, with the increase in the concentration of Poloxamer 407 in the gel, obtained using in addition also HA, the viscosity and strength of the gel increase and the drug was more retained inside the matrix but the release can be conditioned by the presence of ethanol which increase the permeation of cyclosporin A by virtue of its solubility in this solvent. The obtained data following the release of cyclosporin A from the HA-based gel and Poloxamer 407 revealed that the drug was present in the SC at 15% and in the epidermis-dermis layer at 12% after 10 h. Then, the amount of the polymer, cross-linker and drug, as well as the amount and type of absorption promoter can influence drug release from gel formulations in the topical administration of cyclosporin A in the treatment of psoriasis skin lesions, ensuring an adequate concentration of the drug at the skin level and a simultaneous reduction in the systemic absorption of cyclosporine. Furthermore, they could reduce the inflammation affecting the skin and the dermis in the presence of psoriasis [33], as shown by the inhibitory capacity that both empty and full SLN exhibit against nitroxide (Figure 14).

**Acknowledgments:** Academic funds from Department of Pharmacy and health and nutrition sciences—Unical (CS), Italy—Department of Excellence-Law 232/2016.

**Conflicts of Interest:** The authors declare no conflict of interest.

### 3.10. Encapsulation of Prostate-Specific Oncolytic Adenovirus Ad [I/PPT-E1A] in a Novel Core-Shell Structure Nanohydrogel for Cancer Immunotherapy

DengSiyuan^1^*IscaroAlessandra^2^MuthanaMunitta^2^CensiRoberta^1^MartinoPiera Di^1^1School of Pharmacy, University of Camerino, Via S. Agostino 1, 62032 Camerino, Italy2Medical School, University of Sheffield, Beech Hill Road, Sheffield S10 2RX, UK*Correspondence: siyuan.deng@unicam.it

Oncolytic virotherapy has widespread applications in clinical trial for several kinds of cancer [34]. However, the human clinical trials of oncolytic adenovirus are limited to the local administration [35]. Especially for metastatic cancer, the systemic administration of encapsulated adenovirus into nano-scaled delivery system has attracted widespread attention. In this paper, a novel core-shell structure nanohydrogel system based on thiolated hyaluronic acid (HA-SH) and vinyl sulfonated poly(HPMAm-lac_1-2_)-PEG-poly(HPMAm-lac_1-2_) triblock copolymer (Trib-sulf) was developed as a carrier for prostate-specific oncolytic adenovirus Ad Ad[I/PPT-E1A].

After the synthesis of Trib-sulf and HA-SH polymer, [36] nanocapsules were prepared by the W/O emulsion method. HA-SH was dissolved in Ad[I/PPT-E1A] PBS as water phase, while Trib-sulf was dissolved in CHCl_3_ supplemented with lecithin (5.0% w/v). The water phase was added dropwise into organic phase and homogenized at 4 °C. The emulsion was kept at 4 °C overnight under gentle stirring, and then purified by washing with chloroform to remove residual lecithin.

A novel adenovirus delivery system was successfully developed composed by a HA-SH core and Trib-sulf shell. The particle size ranged from 439.6 and 387.9 nm for empty and Ad-loaded nanohydrogel, respectively. The zeta potential was negative −38.19 and 31.85 mV, for empty and Ad-loaded nanohydrogel, respectively. The morphology of nanohydrogel was observed by SEM and TEM. It can be seen the nanohydrogel has a smooth surface and core-shell structure. According to the TEM pictures, it can be visually observed the encapsulation of adenovirus into the nanohydrogel. The toxicity of nanohydrogel and adenovirus activity after encapsulation were measured by MTT assay on PC3 prostate cancer cells. The MTT assay showed the adenovirus can still successfully infect the prostate cancer cells after encapsulation.

In conclusion, in this work, we demonstrated the suitability of the developed HA-SH/Trib-sulf core-shell structure nanohydrogel as delivery system for oncolytic adenovirus. To conclude, we demonstrated that the described nanohydrogel system has interesting biofunctional properties and high versatility of processing, being able to be prepared as nano-scaled delivery system for other kinds of oncolytic adenovirus and also other immune compounds in order to contribute to cancer immunotherapy.

**Acknowledgments:** The authors acknowledge receipt of a European Commission funded H2020 MSCA-ETN grant under proposal number 675743 (project acronym: ISPIC).

**Conflicts of Interest:** The authors declare no conflict of interest.

### 3.11. Extracellular Vesicles as Delivery Carriers of Oncolytic Viruses and Therapeutic Agents for Mesothelioma Treatment

KurykLukasz^1^,^2^RinnerBeate^3^MastrottoFrancesca^4^SalmasoStefano^4^CalicetiPaolo^4^GarofaloMariangela^4^*1Targovax Oy, Clinical Science, 00180 Helsinki, Finland2Department of Virology, National Institute of Public Health—National Institute of Hygiene, 00-791 Warsaw, Poland3Core Facility Alternative Biomodels and Pre-clinical Imaging, Medical University of Graz, Biomedical Research, 8010 Graz, Austria4Department of Pharmaceutical and Pharmacological Sciences, University of Padua, 35122 Padua, Italy*Correspondence: mariangela.garofalo@unipd.it

Despite remarkable improvements achieved in cancer treatment modalities, the outcome remains partially ineffective against different cancer types such as malignant pleural mesothelioma (MPM). Therefore, new and most effective treatment modalities are in high demand. The use of oncolytic viruses (OVs), able to selectively infect, replicate in and induce antitumor immune responses is one of the most promising approaches currently investigated worldwide.

Although promising efficacy was observed in pre-clinical and clinical studies, OVs are often administered intra-tumorally (i.t.), thus many solid tumors cannot be treated using this approach. Additionally, the efficacy of such therapies is limited by pre-existing neutralizing antibodies (NAbs), especially when the virus is administered systemically for a wider biodistribution or to reach multiple metastases. To protect OV against NAbs and enhance antitumor efficacy we decided to encapsulate OVs into extracellular vesicles (EV-OV).

EVs are naturally occurring cargo delivery vesicles with the ability to selectively target the tumor tissue originating the vesicles and to deliver a wide variety of macromolecules. We demonstrated that mesothelioma derived-complex EV-OV resulted in more potent anti-neoplastic activity compared to the virus or EVs alone. Encapsulation seems to protect the OV from immune disruption through their encapsulation into EVs. Furthermore, a program of immunogenic cell death was triggered by the treatments (EV-OV, OV) suggesting that mesothelioma derived-EV-Virus formulations, but not EVs alone, were able to counteract the growth of tumor cells and induce immunogenic cell death.

The presented approach might be a way for a systemic delivery of oncolytic viruses and other drug combinations through EVs for the treatment of cancer malignancies, including mesothelioma.

**Acknowledgments:** MINIATURA (2018/02/X/NZ7/00727) funded by National Science Center, Poland (L.K.); EU COST action CA17140 (M.G. & L.K.); 1BWBW/19 funded by National Institute of Public Health—National Institute of Hygiene (NIPH-NIH), Poland (L.K.).

### 3.12. Development of Poly (Lactide-co-glycolide) Nanoparticles Decorated with Hyaluronic Acid and Loaded with Gold for Visualization and Treatment of Osteoarthritis

GigliobiancoMaria Rosa^1^*ZerrilloLuana^2^D’AtriDomenico^3^GarciaJoao P.^4^RidwanYanto^5^ChanAlan^6^CruzLuis J.^2^CensiRoberta^1^MartinoPiera Di^1^1School of Pharmacy, Drug Delivery Division, University of Camerino, 62032 Camerino (MC), Italy2Translational Nanobiomaterials and Imaging (TNI) group, Radiology department, Leiden University Medical Centrum, 2333 ZA Leiden, The Netherlands3Biotechnology and Food Engineering dept, Technion Israel Institute of Technology, 3200003 Haifa, Israel4Department of Orthopedics, Utrecht Medical Center, 3584 CX Utrecht, The Netherlands5Department of Radiology & Nuclear Medicine, Erasmus University Medical Center, 3000 CA Rotterdam, The Netherlands6Percuros B.V., 2333 CL Leiden, The Netherlands*Correspondence: maria.gigliobianco@unicam.it

Osteoarthritis (OA) represents the most common chronic and degenerative disease. Until now, no specific cures for OA are available, but intra-articular (IA) drug delivery could be a very promising treatment [37]. This treatment allows to minimize the amount of drug for local administration and have high potency and minimal side effects. In particular, glucocorticoids and sodium hyaluronate/hyaluronic acid (HA) are the broadly class of compounds used for OA treatment via IA injection. However, the efficacy of these treatments is relatively short, due to the rapid clearance and short residence time of the glucocorticoids (1–2 h) and HA (22–26 h) in the site of action [38]. 

The present investigation aimed to develop innovative nanoparticles (NPs) drug delivery system-based poly (D, L-lactide-co-glycolide) (PLGA) functionalized with sodium hyaluronate, which is used as a ligand to target specific cell receptors (CD44) for OA treatments [39,40]. The PLGA (RG 503H, 39000Da) was activated by DCC coupling and reacted with the complex of sodium hyaluronate (21kDa-40kDa)/dm PEG (dm PEG; 2000Da) in anhydrous DMSO for two days at room temperature. PLGA-HA copolymer was synthesized with a yield of 67%. ^1^H-NMR in DMSO and D_2_O and FT-IR spectrum pointed out the formation of PLGA-HA copolymer. The amount of covalent grafted HA was 75% obtained by CTAB assay. By using PLGA-HA copolymer, NPs were prepared by a double emulsion solvent evaporation method and co-encapsulated with near-infrared dye (NIR) and gold NPs of 20 nm diameter. NIR dye was useful for visualizing the NPs *in vitro* and *in vivo* through molecular imaging, while gold was used as a contrast agent for *in vivo* µCT scan [41,42]. NPs of PLGA-HA showed a particle size around 200 nm, a low polydispersity index and the zeta potential of −23.15 ± 1.67 mV. Human chondrocytes cell line C28/I2 was used for the cell uptake and binding assay of PLGA and PLGA-HA (40 ug/mL) NPs loaded with NIR dye and detected by Odyssey Infrared Imager 9120 (LI-COR) and confocal microscope (Leica, SP8X). The *in vitro* results gained on C28/I2 human chondrocyte cell line revealed that PLGA-HA enhances accumulation at the target site. The *in vivo* imaging system IVIS Spectrum (Perkin Elmer) was used to measure the retention time of PLGA-HA NPs in the knee joint of male C57BL/6Jico 12 weeks old mice. The PLGA-HA NPs retention in the mouse knee joint was successful results showing that the fluorescent signal of NPs was visible after 15 days of NPs IA injection. 

We successfully demonstrated, by *in vitro* and *in vivo* results, the safe use of PLGA-HA NPs for controlled and sustained delivery of anti-inflammatory or painkiller drugs via IA administrations for the treatment of OA.

**Acknowledgments:** The authors thank PhD M. B. Goldring (Research Division, Hospital for Special Surgery, New York, USA), T. Schomann (Department of Otorhinolaryngology and Head & Neck Surgery; Leiden University Medical Center) for the support with fluorescence microscopy images and I. Que (Translational Nano biomaterials and Imaging (TNI) group, Radiology department, Leiden University Medical Centrum, Leiden, The Netherlands) for the support of the *in vivo* experiment. This work was supported by the following European Union project grants: H2020- MSCA-ITN-2014 TargetCaRe (642414), H2020-MSCA-ITN-2015 ISPIC (675743), H2020-MSCA-RISE-2016 CHARMED (734684) and H2020-MSCA-RISE-2017 CANCER (777682). 

**Conflicts of Interest:** The authors declare no conflict of interest.

### 3.13. Biocompatible Conjugates for Ocular Drug Delivery

KickováEva^1^BalassoAnna^2^PrettoChiara^3^MastrottoFrancesca^1^HestJan van^3^UrttiArto^2^SalmasoStefano^1^CalicetiPaolo^1^*1Department of Pharmaceutical and Pharmacological Sciences, University of Padua, via F. Marzolo 5, 35122 Padua, Italy2Faculty of Pharmacy, Division of Pharmaceutical Biosciences, University of Helsinki, Viikinkaari 5 E, 00014 Helsinki, Finland3Department of Biomedical Engineering, Bio-Organic Chemistry, Eindhoven University of Technology, 5612 AZ Eindhoven, The Netherlands*Correspondence: paolo.caliceti@unipd.it

Age related macular degeneration, an eye disease affecting the posterior segment of the eye, requires invasive techniques and repeated intravitreal injections for drug administration [16,43,44]. Reduced administration frequency is a major requirement to ensure a better patient compliance during treatment. We explored Pullulan anti-inflammatory drug conjugates as therapeutic systems to prolong residence time and control drug release in the vitreous after intravitreal administration.

We have screened a few cleavable linkers for the conjugation of a model molecule, Rhodamine, to Pullulan. A hydrazone linker (HY) in Pull-Rh conjugate was selected based on its sustained release of Rh (50% of Rh, over 10 days under conditions mimicking the vitreous). The hydrophobic character of Rh induced the self-assembly of the conjugate into spherical particles with a size of 25 nm that possess a slow diffusivity in vitreous of about 0.02 μm^2^/s derived by multiple tracking analysis. Moreover, the cytotoxicity and uptake studies investigated on Retinal Pigment Epithelial cells (ARPE-19) confirmed the biocompatibility of the carrier and highlighted a strong cellular uptake after 1 h incubation. The HY linker was then used to generate the conjugate with dexamethasone. 10% w/w of Dexa was conjugated to the Pull backbone. A fluorescent version of the conjugate was generated, Pull-BDP-Dexa, 5% w/w of Dexa and 1.3% w/w of BDP. The chemical identity of the conjugates was assessed by a combination of ^1^H NMR, ^13^C NMR, FT-IR, EA and Snyder assay. The conjugates underwent self-assembly into spherical particles with an average size of 320–370 nm (DLS). The morphology and shape of particles were assessed by TEM and NTA. The release study in PBS at pH 7.4 at 37 °C showed about 20% of Dexa release after 24 h. Ongoing studies will elucidate the release profile under vitreous mimicking conditions. Cytotoxicity studies of Pull-Dexa were performed on HeLa and ARPE-19 cells in a range of 5–600 μM concentration of Dexa with Alamar blue assay, which showed the high biocompatibility of the conjugate. Cell uptake studies performed by incubating the same cell lines with Pull-BDP-Dexa for 24 h showed a significant association of the conjugates with cells.

In summary, the synthesis of Dexa and BDP to Pull with HY linker were successfully set up and conjugates underwent an extensive characterization. The conjugates are promising because they possess slow diffusivity in the vitreous in which can anticipate higher residence time. Cytotoxicity and uptake study on ARPE-19 confirm that the conjugates are not toxic and efficient in associating to the cells. The future investigations will quantify cell association of the Pull-BDP-Dexa with cytofluorimetric analysis and confocal microscopy. Defined conditions mimicking the vitreous will be used to assess diffusivity of Dexa loaded particles by NTA.

**Acknowledgments:** This project has received funding from the European Union’s Horizon 2020 research and innovation program under the Marie Skłodowska-Curie grant agreement N° 722717.

**Conflicts of Interest:** The authors declare no conflict of interest. The founding sponsors had no role in the design of the study; in the collection, analyses, or interpretation of data; in the writing of the manuscript, and in the decision to publish the results.

### 3.14. GMP-Compliant Freeze-Dried Extracellular Vesicles: Regenerative and Nano-Drug Delivery Systems

PerteghellaSara*BariEliaTorreMaria LuisaDepartment of Drug Sciences, University of Pavia, Viale Taramelli 12, 27100 Pavia, Italy*Correspondence: sara.perteghella@unipv.it

Mesenchymal Stem Cells (MSCs)-secretome is composed of soluble factors (proteins, growth factors, and cytokines) and extracellular vesicles (EVs) that can be divided into microvesicles and exosomes. MSC-secretome represents a valid therapeutic approach in place of parental cells for the treatment of chronic and acute diseases [45]. Despite the great interest addressed to the MSC-secretome, missing steps are required for the secretome “pharmaceuticalization” to obtain a high quality, safe and effective medicinal product, as well as for the optimal formulation development.

Important requirements for the development of a medicinal product are: To obtain a scalable and GMP-compliant production process and the stability and the shelf-life of the final product; ultrafiltration, as purifying process, and freeze-drying fit with this requirement. We combined these two techniques obtaining a ready-to-use powder named lyo-secretome [46,47]. EV integrity and morphology were not affected by the technological processes; product resulted as no cytotoxic and no hemolytic confirming its safety profile. Efficacy of lyo-secretome was proven against oxidative stress-induced damages [2], as immunomodulant agent [47] and in a wound healing murine model. Furthermore, proteomic characterization of lyo-secretome revealed that it contains alpha1-antitripsin (AAT) with in vitro anti-elastase activity, paving the way for the use in lung degeneration AAT deficiency [48].

All this evidence demonstrated that lyo-secretome could be considered to be a new active pharmaceutical ingredient (API). Furthermore, the nano-size and the membrane structure of EVs make them ideal candidates as nano-drug delivery systems (DDS); EVs resulted as similar to liposomes but, at the same time, presented high stability in bloodstream and low immunogenicity with respect the synthetic nanosystems. MSC-EVs maintained the homing ability to injured site of their parental cells; this characteristic makes MSC-EVs suitable smart DDS to avoid off side effects, and enhance the specific uptake by target cells. In this context, we proposed the combined use of silk fibroin nanoparticles, as technological carrier, and EVs, as biological carrier, to obtain a drug delivery system named “carrier in carrier”. The nanoparticles can efficiently incorporate hydrophobic and/or hydrophilic drugs, while the EVs could assure an adequate drug targeting due to their innate ability to reach the damaged tissues.

This combined biological-technological approach could represent a novel class of nanosystems, combining beneficial effects of both regenerative cell therapies and pharmaceutical nanomedicine, avoiding the use of viable replicating stem cells [49]. This evidence demonstrates that MSC-secretome can be used both as API and DDS; although human and veterinary clinical trials are still few, we are confident that many will start in the nearly future.

**Acknowledgments:** This work was supported by Interreg V-A Italy-Switzerland 2014-2020-ATEx-Advanced Therapies Experiences. Project ID 637541.

### 3.15. Cell Internalization Kinetics of Biodegradable Nanoparticles Decorated with Hyaluronic Acid

SilvestriTeresa^1^MayolLaura^1^*BorzacchielloAssunta^2^SalaFrancesca della^2^BiondiMarco^1^1Dipartimento di Farmacia, Università di Napoli Federico II, 80138 Napoli, Italy2Consiglio Nazionale delle Ricerche (CNR), Istituto per i Polimeri, Compositi e Biomateriali (IPCB), 80125 Napoli, Italy*Correspondence: laumayol@unina.it

Among the strategies to endow nanodevices with active tumor targeting ability, their surface decoration with hyaluronic acid (HA) indisputably plays a major role. Indeed, HA is a negatively charged polysaccharide possessing a strong tropism toward solid tumors due to preferential interaction with CD44 and RHAMM receptors, which are overexpressed in a wide array of cancer cells [50,51]. Consequently, nanocarriers exposing HA on their surface may be endowed with a specific recognition ability toward tumors [52]. In previous studies, it has been suggested that HA-coated nanoparticles (NPs) can be internalized by a receptor-mediated mechanism [53]. Thus, the main hypothesis of this work is that the mode of ligand presentation can determine the cellular uptake of NPs. Here, poly(lactic-co-glycolic acid) (PLGA)-based nanoparticles (NPs) have been decorated with HA without chemical reaction, using amphiphilic poloxamers (namely F68 and F127) as bridging moieties between hydrophobic PLGA and hydrophilic HA. Two HA molecular weights were chosen (200 and 800 kDa; NP formulations were named HA2 and HA8, correspondingly) and kinetic internalization studies were carried out on breast carcinoma (HS578T) and healthy mouse fibroblast (L929) cells, used as a control.

NPs were produced by a modified nanoprecipitation technique, by forcing a PLGA/F68/F127 solution in acetone (1:0.5:0.5 weight ratio; 5 mL, 3% w/v) through a needle of a syringe at 333 µL/min by a Syringe Pump. The solution was precipitated into 40 mL of an aqueous phase (W1) containing F127 and F68 as surfactants (1:1 w/w ratio; 0.05% w/v) and HA (3.75 and 0.81 mg/mL for HA2 and HA8 NPs). The organic solvent was evaporated overnight, and the obtained NPs washed twice times by centrifugation (10,000 rpm, 20 min) and stored at −80°C. NPs were characterized for their morphology by TEM, while size and z-potential (ZP) by PCS. NP architecture was studied by differential scanning calorimetry (DSC) (10–80 °C, 5 °C/min, under inert nitrogen atmosphere). Cellular uptake kinetics of fluorescent NPs, prepared by adding Nile Red (NR) to the organic phase (0.01% w/w NR:polymers), were obtained by performing, at scheduled time points, cell lysis followed by a spectrofluorimetric assay on the lysate to quantify NP-associated fluorescence.

**Figure 15:**
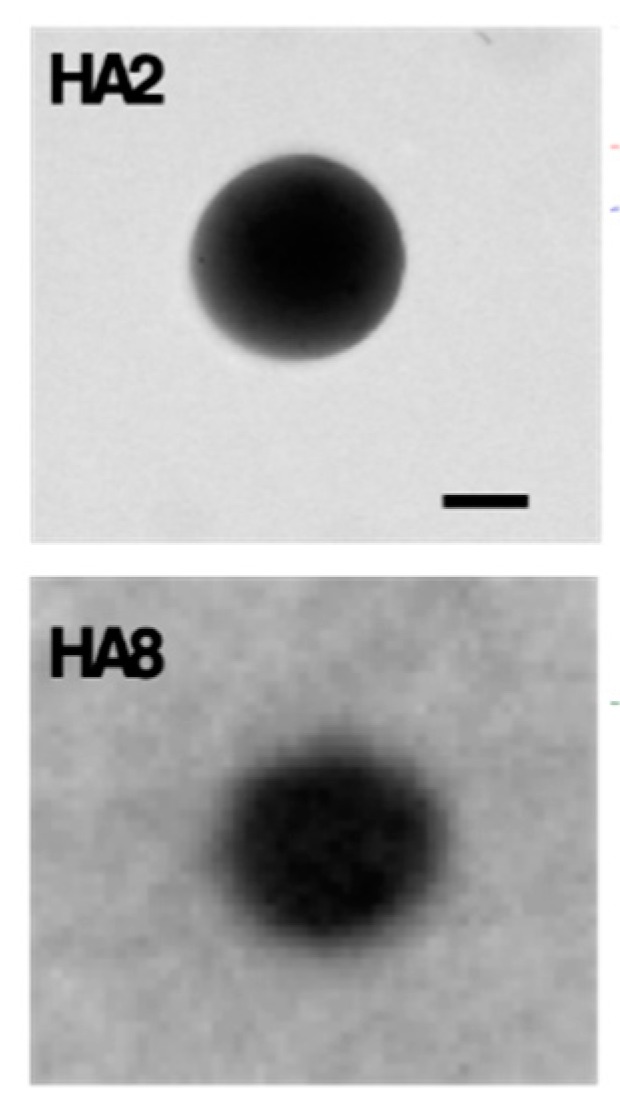
TEM images of HA2 and HA8 NPs.

Spherical NPs with a <200 nm mean size was obtained. ZP values are <−40 mV, indicating the successful HA arrangement on NP surface (Figure 15). DSC results indicated that, for HA2 and HA8 NPs, melting temperature (Tm) and heat (ΔHm) are lower compared to Tm and ΔHm of poloxamers alone, indicating the loss of crystalline poloxamer regions in rubbery PLGA matrix and the formation of a co-amorphous, partially phase-separated system in which the polymers partially behave as separate entities (Table 1). 

**Figure 16:**
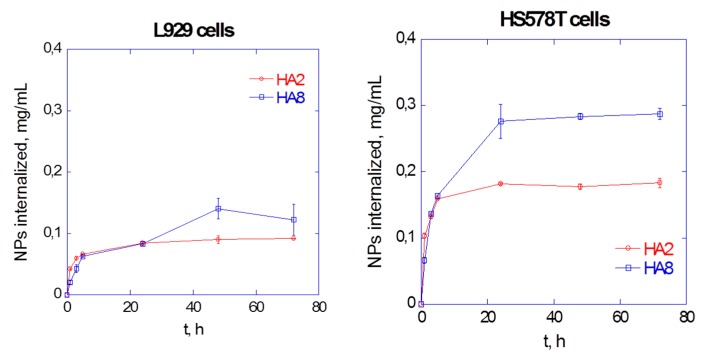
Uptake kinetics of HA2 and HA8 NPs.

Results of cell internalization showed that both HA2 and HA8 NPs enter more effectively in CD44-overxpressing tumor cells than in healthy cells, indicating that HA-containing NPs do possess a tropism for HS578T cells. More in detail, the uptake of HA8 NPs is higher than HA2 NPs in both cell lines (Figure 16), thereby hinting at a different molecular arrangement of high molecular weight HA which in turn affects the precocity of interaction between HA-decorated nanodevices and the target cells.

In conclusion, in this work PLGA-based NPs have been successfully decorated with HA and their tropism for CD44-overexpressing cells has been shown. Moreover, HA8 NPs showed a higher uptake propensity than HA2 NPs.

### 3.16. Astaxanthin Loaded Stealth Solid Lipid Nanoparticles (S-SLN) Interact with Biomembrane Models: Calorimetric Evidence

SantonocitoDebora*PugliaCarmeloSarpietroMaria GraziaCastelliFrancescoDepartment of Drug Sciences, University of Catania, 95125 Catania, Italy*Correspondence: debora.santonocito@outlook.it

Lipid nanoparticles have revolutionized the release of drugs. Their systemic use is limited by the presence of the Mononuclear Phagocytic System and the Reticuloendothelial System; therefore, to increase the bioavailability of nanoparticles it is necessary to modify their surface with polyethylene glycol (PEG) to realize “stealth” systems (S-SLN) [54,55]. The polymer fits into the surface of the nanoparticle giving it an umbrella structure. This steric encumbrance causes a delay of attack by macrophages, therefore, this strategy increases intracellular bioavailability of the drug and leads to better therapeutic efficacy. The aim of this research is to find an efficient formulation strategy for obtaining stable and homogeneous S-SLNs loaded with astaxanthin (AST), a natural powerful antioxidant, for the treatment of Alzheimer’s disease through parenteral administration and to study the interaction between biomembrane models and S-SLN to get information on the mechanism by which the systems interact with biological membranes.

SLN were prepared using a slightly modified solvent-diffusion technique. Stearic acid and DSPE-PEG2000 were solubilized in ethanol (70 °C) and the mixture was stirred to obtain a dispersion. This lipid phase was added to the hot aqueous phase (hydroxypropyl-methyl cellulose, soy lecithin, Poloxamer 188), afterwards the mixture was emulsified at 13500 rpm, 70 °C for 8 min, subjected to ultrasonication for 10 min and then let cooling at 4 °C. The loaded SLN were prepared with the addition of AST (1 mmol). The average particle size and the polydispersity index of the SLN were measured. MLV, used as biomembrane model, were prepared using the thin-layer evaporation method [56]. Dimyristoylphosphatidylcholine was dissolved in an organic solvent mixture. The solvents were evaporated, and the samples were lyophilized. The resulting phospholipid films were hydrated using Tris solution. The samples were mixed 1 min and kept a 37 °C 1 min, for three times and, finally, were kept at 37 °C for 1 h. Differential scanning calorimetry was used to study the thermotropic behavior of S-SLN and to evaluate, by kinetic experiments, the interaction occurring between S-SLN and MLV at increasing contact time. Analyses were carried out in the range 5–85 °C.

Experimental data indicated that nanoparticles had a mean size of about 125 nm and a polydispersity index of 0.244. DSC analysis on SLN indicates a stabilization of the SLN structure in the presence of PEG and a synergistic effect of AST and PEG on SLN. Kinetic experiments on MLV and S-SLN kept in contact at increasing incubation time, showed a gradual variation of the calorimetric curves of S-SLN and MLV indicating that S-SLN entry into the MLV and lose their integrity with the release of AST inside the MLV.

Our results suggest that these S-SLN could be regarded as a promising drug delivery system to improve AST bioavailability and antioxidant activity.

### 3.17. Design of Alginate Based Microcapsules for Delivering of Probiotic Bacteria as Functional Supplements in Fruit Juice 

BonaccorsoAngela^1^*RomeoAlessia^1^RussoNunziatina^2^GrimaudoMaria Aurora^3^Alvarez-LorenzoCarmen^3^CarboneClaudia^1^RandazzoCinzia L.^2^CaggiaCinzia^2^PuglisiGiovanni^1^MusumeciTeresa^1^1Department of Drug Sciences, University of Catania, 95125 Catania, Italy.2Department of Agriculture, Food and Environment, University of Catania, 95123 Catania, Italy.3Department of Farmacología, Farmacia y Tecnología Farmacéutica, R+DPharma Group (GI-1645), Facultad de Farmacia and Health Research Institute of Santiago de Compostela (IDIS), Universidade de Santiago de Compostela, 15782 Santiago de Compostela, Spain*Correspondence: abonaccorso@unict.it

Presently, the interest in “functional foods” has increased in the general population due to their positive effect on human health, beyond their basic nutrition value. These products contain bioactive compounds such as fibers, oligosaccharides or probiotics whose consumption can lead to maintain good health [57]. Many foodstuffs containing probiotics or prebiotics are milk products, but alternatives are emerging for people who cannot consume dairy products (i.e., lactose intolerant or consumers with hypercholesterolemia). Fruit juice may represent a valid probiotic vehicle thanks to its high healthy nutritional value and the large use by all age consumers. To increase the survival of probiotics during production processes and their stability in acidic matrices we aimed to design and characterize a formulation based on alginate microcapsules (MCs) containing probiotic bacteria. In particular, MCs were designed and optimized by using Box Behnken design [58]. A total of 17 MCs formulations were prepared by ionotropic gelation method and characterized for their mean size, polydispersity and surface charge by using a Zetasizer Nano ZS90 (Malvern Instruments). The selected MCs formulation was optimized based on the desirability function (0.973) and loaded with the *Lactobacillus rhamnosus* GG (LGG) strain. The loaded MCs were analyzed by fluorescence microscopy and collected at regular intervals to evaluate the strain viability by count plate method on MRS agar incubated at 37 °C for 48 h. Loaded MCs with probiotic cells were uniform and homogeneous (P.D.I. <0.3), with a slight increase in diameter (~1 µm), compared to the empty MCs. The MCs were also investigated for morphology by using a high-resolution transmission electron microscope (JEM-1011, JEOL USA Inc., Peabody, MA, USA). Results on stability studies revealed the strain survival in the final product at 5 °C until 28 days. The addition of microencapsulated LGG on fruit juice did not significantly affect the rheological properties of juice based on a macroscopic observation. Therefore, further studies are ongoing to investigate the efficacy of microencapsulation to deliver probiotics in a controlled manner to the gastro-intestinal tract.

### 3.18. Localized Treatment of Nasal Wounds Using a Thermosensitive Chitosan-Based Nasal Spray

GholizadehHanieh^1^,^2^MesserottiElisa^2^,^3^PozzoliMichele^2^ChengShaokoon^1^TrainiDaniela^2^YoungPaul^2^KourmatzisAgisilaos^4^CaramellaCarla^3^OngHui Xin^2^1School of Engineering, Macquarie University, Sydney NSW 2109, Australia2Respiratory Technology, Woolcock Institute of Medical Research, Discipline of Pharmacology, Faculty of Medicine and Health, Sydney NSW 2050, Australia3Department of Drug Sciences, University of Pavia, 27100 Pavia, Italy4School of Aerospace, Mechanical and Mechatronic Engineering, The University of Sydney, Sydney NSW 2006, Australia*Correspondence: carla.caramella@unipv.it

Loss of integrity of nasal mucosa may be accompanied by in nose bleeding (epistaxis), which may require medical intervention to control hemorrhage [59]. Available medications may cause discomfort and complications [60].

The aim of the work was to prepare a nasal spray formulation endowed with in situ gelling properties. A thermosensitive nasal formulation based on chitosan (CS), and β-glycerophosphate (GP) salt, was prepared (by mixing the CS and CP solutions in an optimized volume ratio) and loaded with tranexamic acid (TXA), one of the most effective pharmacological tools to control bleeding, at different concentrations [61,62].

**Figure 17:**
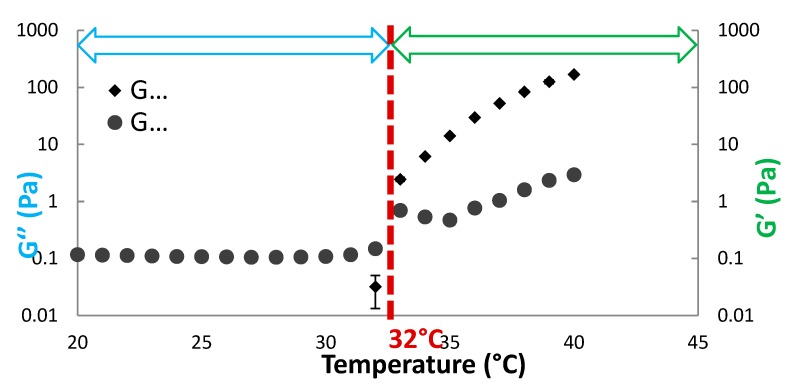
Representation of gelation temperature.

**Figure 18:**
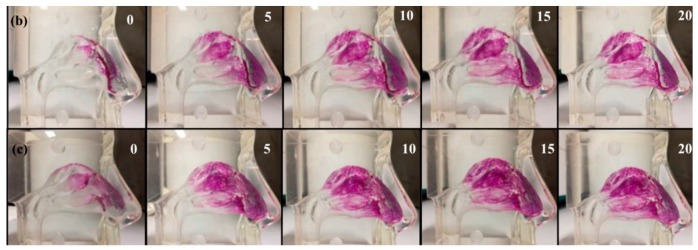
The gelation of the formulation (**b**) on the nasal surface (human nasal model) occurred in 5 min at 37 °C and stopped nasal run off, and the deposition pattern on the wall of the nasal cast cavity remained unchanged from 5 to 20 min. In contrast, control continued to show leakage (**c**). The aerodynamic particle size distribution (about 90% >10 µm) was consistent with nasal deposition.

The thermogelling properties, in terms of gelation time and temperature, were investigated using both the inverted test tube method and rheology. The reversibility of sol–gel transition was also assessed. The spray and aerosolization properties of the nasal formulation were investigated and characterized in terms of spray pattern (visually) nasal deposition (using a human nose model), aerodynamic particle size distribution and deposition pattern in the respiratory tract (using the USP Next Generation Impactor). The following in vitro biological tests were performed: cytotoxicity (using a viability test on RPMI 2650 epithelial nasal cells) and wound healing properties (using a scratch assay on nasal cells on an Air Liquid Interface model). Main results are shown in Figure 17 and Figure 18.

The intersection between G” and G’ at 32 °C indicated the gelling temperature (Figure 17). The sol–gel transition was reversible until the third cycle of heating and cooling. The approximate gelation time for TXA-CS-β-GP with 1% w/v TXA was 5 min [63].

**Figure 19:**
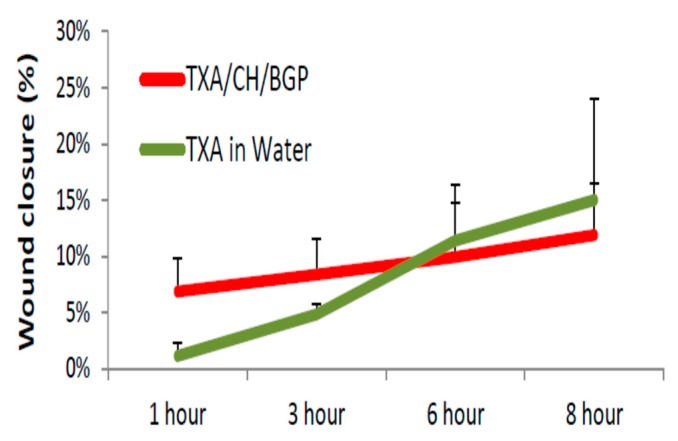
In vitro cell migration (expressed as % closure of the gap versus time) in scratched nasal epithelium cell layer: (red) formulation, (green) control.

The developed formulation showed no toxic effect on human nasal epithelial cells at the concentrations used and allowed faster wound gap closure as compared to the control TXA solution. The onset of wound closure was 5.94 times faster (at 1 h) after application of the formulation as compared to the control (Figure 19).

In conclusion, the nasal spray developed showed rapid gelation in 5 min at 32 °C with reversible mechanism and an ideal spray and deposition pattern for nasal drug delivery. It could prevent nasal runoff and enhance residence time. It proved safe on human nasal epithelial cells and efficient in wound closure in the first 3 h.

**Acknowledgments:** The support of the II level Master Course in Pharmaceutical Technology and Regulatory Affairs of Pavia University is greatly appreciated.

**Conflicts of Interest:** The authors declare no conflict of interest. Figure 17 and Figure 18 are modified from a related paper submitted to AAPS Pharm Tech Journal (http://dx.doi.org/10.1208/s12249-019-1517-6). 

### 3.19. Contact Lenses with Bioinspired Receptors for Naltrexone, an Antidiabetic Drug

Alvarez-RiveraFernando^1^SerroAna Paula^2^,^3^SilvaDiana^3^Alvarez-LorenzoCarmen^1^ConcheiroAngel^1^*1Departamento de Farmacología, Farmacia y Tecnología Farmacéutica, I+D Farma (GI-1645), Facultad de Farmacia and Health Research Institute of Santiago de Compostela (IDIS), Universidade de Santiago de Compostela, 15782 Santiago de Compostela, Spain2Centro de Investigação Interdisciplinar Egas Moniz, Instituto Universitário Egas Moniz, Quinta da Granja, Monte de Caparica, 2829-511 Caparica, Portugal3Centro de Química Estrutural, Instituto Superior Técnico, Universidade de Lisboa, Av. Rovisco Pais, 1049-001 Lisboa, Portugal*Correspondence: angel.concheiro@usc.es

Diabetes causes relevant metabolic changes that trigger ocular surface complications such as decreased tear production, diminished corneal sensitivity, and delayed wound healing. The natural opioid growth factor (OGF) enkephalin has been found to be at higher levels in persons with diabetes compared to healthy people, causing increased susceptibility to ulceration and infection due to delayed epithelialization during wound healing. Thus, antagonists of its receptor (OGFr), such as naltrexone (NTX), may revert enkephalin-dependent ocular surface complications [64]. Repeated doses of NTX as eye drops have been shown to be safe, but poorly efficient. Thus, the aim of this work was to design hydrogels suitable as soft CLs that can load therapeutic amounts of NTX and provide sustained release on the ocular surface.

Poly(2-hydroxyethyl methacrylate) (pHEMA) hydrogels were endowed with affinity for NTX by mimicking the functional groups and conformation of OGFr. Several hydrogels were prepared with functional monomers bearing carboxylic acid groups (acrylic acid, AAc) and aromatic groups (benzyl methacrylate, BzMA). It was hypothesized that the addition of NTX before polymerization may drive the adequate spatial arrangement of the monomers for a more efficient formation of ad hoc artificial receptors (imprinted hydrogels) [65]. Both imprinted and non-imprinted hydrogels were washed and then loaded by soaking in NTX aqueous solutions. Imprinted hydrogels containing AAc mers loaded up to 10.83 ± 0.38 mg NTX/g. Feasibility of carrying out loading and sterilization (autoclaving) processes in one step was confirmed. NTX-loaded hydrogels were highly cytocompatible with human mesenchymal stem cells (hMSC) and passed the HET-CAM test.

NTX release was recorded using both a conventional test in bulk medium (sink conditions) and a microfluidic device mimicking lachrymal fluid turn over [65]. The bioinspired hydrogels showed sustained release for 24–48 h under sink conditions and much more prolonged release when the microfluidic chamber was used. Interestingly, the hydrogels with higher affinity for NTX in the loading test were also the ones that sustained better the release in any setup used. Finally, ex vivo corneal tests revealed that NTX mainly accumulated into the cornea at therapeutic levels, with minor permeation to the receptor compartment simulating the aqueous humor. Thus, NTX-eluting hydrogels may represent a promising tool to locally address eye complications of diabetes mellitus.

**Acknowledgments:** This work was supported by MINECO [SAF2017-83118-R], Agencia Estatal de Investigación (AEI) Spain, Xunta de Galicia [ED431C 2016/008] and FEDER.

### 3.20. Lipid-Based Nanoparticles for Cutaneous Tocopherol Delivery

SguizzatoMaddalena^1^,^2^EspositoElisabetta^1^*HallanSupandeep Singh^1^DrechslerMarkus^3^ValacchiGiuseppe^2^CortesiRita^1^*1Department of Chemical and Pharmaceutical Sciences (SCF), University of Ferrara, 44121 Ferrara, Italy2Animal Sciences Department, NC State University, Plants for Human Health Institute, Kannapolis, NC 27695, USA3BIMF/Soft Matter Electron microscopy, University of Bayreuth, 95447 Bayreuth, Germany*Correspondence: ese@unife.it (E.E.); crt@unife.it (R.C.)

Recently many antipollution dermocosmetics have been produced with the aim to defend the skin against prolonged and repetitive daily exposure to pollutants. Nevertheless, this strategy offers a short-term improvement of skin barrier function. In this respect there is a need for an efficacious product, endowing skin protection from pollutants in long-term exposure, as well as for antipollution test methods suitable for assessing product efficacy and safety [66].

This study focuses on the production of lipid nanoparticles for cutaneous antioxidant delivery. Indeed, several molecules, such as α-tocopherol have been shown to improve skin condition and even counteract the effects of exogenous challenges such as smoking on skin ageing. Particularly this work describes the design and development of lipid nanoparticles containing α-tocopherol as antioxidant agent aiming at protection of the human skin against pollutants. Specifically, solid lipid nanoparticles (SLN) and nanostructured lipid carriers (NLC) were prepared using different lipids (tristearin, Compritol^®^, Precirol^®^ or Suppocire^®^) in the presence or in the absence of caprylic/capric triglycerides. The formulations were characterized in terms of size, morphology, encapsulation efficiency, in vitro cytotoxicity and protection against cigarette smoke.

As compared to SLN, NLC enabled a reduction on the agglomerate formation and a control on size stability, suggesting their suitability for antioxidant loading. Antioxidant encapsulation efficiency was evaluated by HPLC upon disaggregation of nanostructured lipid carriers.

The external and inner structures of α-tocopherol-containing NLC were analyzed by cryogenic transmission electron microscopy and X-ray spectroscopy, respectively. Apart from Suppocire^®^, leading to formation of spherical vesicles, the other lipids resulted in irregular shaped nanoparticles. A doubling in the lipid phase amount enabled a doubling of the loading within the nanoparticles, controlling the drug stability up to 3 months.

Tristearin-based nanostructured lipid carriers loaded with α-tocopherol were selected for ex vivo studies since they displayed better physico-chemical properties as compared to the other NLC compositions. Human skin explants were treated with α-tocopherol loaded nanostructured lipid carriers and then exposed to cigarette smoke, afterwards protein levels of the stress inducible enzyme heme oxygenase were analyzed in skin homogenates. Interestingly, it was found that the pretreatment avoided heme oxygenase up-regulation, suggesting a protective effect of the nanoparticles.

**Acknowledgments:** This work was funded by “FIR 2018” and “FAR 2018” of Ferrara University, Italy. No funds were received to cover the costs to publish in open access.

### 3.21. Colon Delivery of Nutraceutical Ingredients Using Food-Grade Polymer-Based Microparticles

CurcioClaudia^1^CorsaroRoberta^1^BonaccorsoAngela^1^MusumeciTeresa^1^RuoziBarbara^2^PignatelloRosario^1^*1Department of Drug Sciences, University of Catania, 95125 Catania, Italy2Department of Life Sciences, University of Modena and Reggio Emilia, 41121 Modena, Italy*Correspondence: r.pignatello@unict.it

Nutraceuticals are naturally occurring substances defined as nutritional components that induce physiological or therapeutic benefits able to prevent the onset of chronic diseases. Compared to pharmacological treatments, they have an intrinsically reduced toxicity and generate less side effects. Nutraceuticals include lipids, vitamins, phenolic compounds, peptides, and other compounds isolated from various sources such as spices, herbs and fruit. Oral administration of nutraceuticals is preferred in the case of chronic diseases, because it follows the same natural processes of food and nutrient intake in the body, being a non-invasive and high-compliance route.

However, the limited solubility of most nutraceuticals, as well as the conditions of the gastro-intestinal tract (GIT) to which they are exposed, such as the low pH in the stomach, or presence of degradative and metabolic enzymes in the gut, largely reduce their overall bioavailability and therefore the therapeutic efficacy. In this respect, delivery systems, already exploited for the controlled and targeted oral release of drugs, become to be applied also in the nutraceutical field with some success [67]. In particular, conditions such as inflammatory bowel diseases (IBD), including ulcerative colitis and Crohn disease, as well as colorectal cancer would benefit of a specific colon delivery of active ingredients, without a systemic adsorption during the transit of the gut.

pH-dependent drug release is one of the strategies proposed to focalize the delivery of actives within the colon, taking advantage of the relatively higher pH value of this area compared to the previous GIT sections [68]. The aim of this study was the production of polymeric microparticles for the controlled intestinal release of natural compounds, with adjuvant activity in the treatment of IBD and other colon diseases.

In particular, we chose a class of acrylate polymers, commercialized as Eudraguard^®^ by Evonik (Germany); they are included in the EU food additive list and received GRAS status in the USA. As well as the analogous ‘pharma-grade’ Eudragit^®^ copolymers, Eudraguard^®^ have been essentially proposed by the manufacturer for coating solid oral dosage forms, to achieve a pH-dependent controlled release. In previous works of ours, Eudragit^®^ RS100 and RL100 matrices have been instead investigated for producing micro- and nanosized systems for the modified delivery of drugs and natural ingredients [69,70,71,72]. We therefore hypothesized that also Eudraguard^®^ polymers, and in particular Eudraguard^®^ Control (EUG-C) and Biotic (EUG-B) could be suitable ingredients to produce microparticulate systems for the pH-controlled oral delivery of bioactive compounds.

EUG-C is a neutral copolymer based on ethyl acrylate and methyl methacrylate with a ratio of 2:1. Coating with this material can protect nutraceutical actives from the acidic environment of the stomach. EUG-B is an anionic methacrylate copolymer based on methacrylic acid, methyl methacrylate and methyl acrylate. The ratio of the free carboxyl groups to the ester groups is approx. 1:10. Being soluble at pH values above 7.0, it can be used as a pH-triggered polymer to protect compounds from gastric acid in the stomach and bile acid in the small intestine, ensuring a release once the systems reach the colon.

We used EUG-B and EUG-C, alone or in combination, to produce microparticles loaded with quercetin and resveratrol as models of a low-solubility and a high-solubility active compound, respectively. Different production techniques were investigated to optimize yields and quality of the microparticles. The microcarriers were then tested to verify the ability of these copolymers, in the form of a matricidal system instead than as a coating layer, to limit the dissolution of the actives in the stomach and to control their release at the various intestinal pH values.

**Acknowledgments:** Rofarma Italia srl is gratefully acknowledged for providing Eudraguard products.

### 3.22. Biodegradable Nanoparticles for Ophthalmic Diseases

GaetanoFederica De*VenturaCinzia AnnaDepartment of Chemical, Biological, Pharmaceutical and Environmental Science, University of Messina, V.le F. Stagno D’Alcontres, 98166 Messina, Italy*Correspondence: fedegaetano@unime.it

The bacteria can form ocular biofilms. The biofilm has been extensively studied by Costerton and his research group [73]. Briefly, a biofilm can be defined as a colony of microorganisms, in which they are attached to each other and generally have the ability to adhere to a surface, such as the eye. Biofilm formation gives individual bacteria the ability to collaborate and adapt to difficult environmental conditions. There is growing evidence that biofilms are the cause of different types of eye infections, many of which are resistant to antibacterial treatment [73]. Presently, antibiotic resistance represents a problem for world health, because it causes persistent infections and mortality [74]. Treatment with the free drug often requires high toxic doses for the patient, with serious or deadly side effects [75]. A novel anti-biofilm strategy is needed.

In this study, we designed chitosan nanoparticles (CH-NPs) loading levofloxacin (LEF) for the treatment of ophthalmic diseases. LEF is an antibiotic active towards different types of ocular infections. The ophthalmic aqueous formulation of LEF has been approved in Europe, USA and Japan. However, this formulation could not be sufficient to penetrate bacterial biofilms and consequently the treatment with free LEF may not have therapeutic efficacy. Chitosan (CH) is a cationic polysaccharide, widely used in the medical and pharmaceutical fields due to its high biocompatibility, biodegradability, mucoadhesive ability and non-toxicity. It also shows good antibacterial properties. In this study, CH-NPs were prepared by ionotropic gelation method that exploits the electrostatic interaction between positively charged CH and negatively charged gelling agents. Different types of polyanions were used that are sodium tripolyphosphate (TPP) or sulfobutylether-β-cyclodextrin (SBE-β-CD). Low molecular weight CH was solubilized in 1% acetic acid solution, after brought to pH 5. Aqueous solutions of different polyanions were added dropwise to the CH solutions under continuous magnetic stirring for 30 min. The colloidal suspensions were centrifuged twice at 13000 rpm for 30 min, to allow optimal separation of the pellet from the supernatant. The pellets were re-suspended in 1 mL of water containing trehalose (5%, w/v) as a lyoprotectant agent and finally freeze-dried. The supernatants were collected and used for determination of encapsulation efficiency (E.E.) of LEF. Morphology of the CH-NPs was investigated by scanning transmission electron microscopy (STEM). We obtained spherical and small CH-NPs, with sizes between 250 and 350 nm, which showed positive zeta potential values, these needed for the interaction of the NPs with the biofilm negatively charged. STEM images demonstrated for the CH-NPs gelified with TPP (CH-NPs/TPP) homogeneous morphology, instead NPs prepared with SBE-β-CD showed a dense core surrounded by a less dense shell probably made of not gelified CH chains. CH-NPs/TPP showed higher yield percentage and EE percentage with respect to CH-NPs/SBE-β-CD; however, the drug content is higher for these latter NPs. This could suggest the presence in solution of a complex between LEF and the macrocycle. To clarify this hypothesis, we investigated the complexation by using UV-Vis and 1H-NMR spectroscopy. A 1:1 interaction between LEF and the CD was revealed. In vitro release of LEF from CH-NPs was determined using dialysis membranes, in the absence and presence of lysozyme, this presents in the eyes at high concentrations. In all cases the presence of lysozyme increased the amount of LEF released within 24h.

In conclusion, LEF can be efficiently encapsulated in CH-NPs. Biological in vitro and ex vivo studies on ocular keratitis models are in progress to evaluate the potentiality of CH-NPs loading LEF for the treatment of ophthalmic diseases.

### 3.23. Development of Propranolol Hydrochloride Loaded EUDRAGIT^®^ EPO Microbeads Obtained with Prilling Technique for Pediatric Oral Administration

LopalcoAntonio^1^DenoraNunzio^1^LaquintanaValentino^1^CutrignelliAnnalisa^1^FrancoMassimo^1^RobotaMiriam^2^HauschildtNina^2^MondelliFrancesco^1^ArduinoIlaria^1^LopedotaAngela^1^*1Department of Pharmacy-Pharmaceutical Sciences, University of Bari “Aldo Moro”, 70121 Bari, Italy2Formulation and Application Services, Evonik Nutrition & Care GmbH, 45128 Darmstadt, Germany*Correspondence: angelaassunta.lopedota@uniba.it

The aim of this work was the development and optimization of new pediatric solid formulations of the active pharmaceutical ingredient (API) propranolol hydrochloride (PR), used to treat various pediatric diseases including hemangioma.

PR has a bitter salty taste that does not permit a good compliance of children especially in liquid formulations. In addition, solid formulations, such as tablets, are aimed for adults and often their dosage is not suitable for young patients that require a flexible dose based on their weight. Therefore, to overcome these limitations microbeads based on EUDRAGIT^®^ EPO, a taste masking excipient, containing PR were formulated using the innovative prilling-congealing technique.

Different formulations were prepared and characterized in terms of particle size, drug loading, efficiency of encapsulation. Their morphology was evaluated by scanning electron microscope (SEM). Solid-state of the API in the obtained microbeads was investigated by Fourier transform infrared spectroscopy (FT-IR) and differential scanning calorimetry (DSC) analyses. The in vitro drug release and electronic tongue (e-tongue) tests of the formulations were also performed in order to evaluate the efficacy of these microparticulate systems in avoiding the bitterness in the mouth after administration and the complete release of the API in the stomach.

Using nozzles 300 and 450 μm (code n), the diameters of the microbeads ranged from 333 to 699 μm and appeared homogenous and appropriate to be swallowed by children. The ratio drug/matrix for the microbeads was also studied in detail: 1:25 (A_1_), 1:15 (A_2_) and 1:10 (A_3_) in pure water and tert-butyl alcohol/water (code t) mixture. Most of the examined microbeads were characterized by high percentage of encapsulation efficiency (between 81 and 100%) and drug loading (between 22 and 77 mg of drug per g of matrix), effective for the administration of low and high doses of the API. SEM analysis revealed a matrix with numerous pores and a radial or spongy structure. Release studies confirmed a low release/dissolution of the API in artificial saliva (pH 5.5 and 6.8), mainly A_1n_ > A_1_ > A_2nt_, and a prompt dissolution in simulated gastric media (pH 1.2 and 4.0). Finally, e-tongue measurement confirmed the ability of the microbeads to mask the bitter drug taste especially for the sample A_1n_. This last sample and A_1_ were chemically and physically stable for four months.

In conclusion, the optimized microbeads A_1_ and A_1n_ reached the goal of this study and could be proposed as new solid oral formulations dedicated to young patients. These multiple-unit systems could prove to be better than single-unit systems due to their advantages: flexible dose, better patience compliance by masking an unpleasant taste, and less swallowing and stability problems. Furthermore, these flexible modified dosage forms could comply with prescribed dosing regimens due to differences in age and weight of child.

**Acknowledgments:** We want to thank Evonik Nutrition & Care GmbH from Evonik for the performance of SEM analyses and financing the e-tongue measurements and Pasquale Trotti (in charge of the electronic microscopy service at the department of Entomology and Zoology section of DISSPA UNIBA) for his advices and contribution for SEM analysis as well.

### 3.24. Coating of Retentive Drug Delivery Systems Based on Shape-Memory Poly(vinyl Alcohol) for Improved Release Performance

UboldiMarco*MelocchiAliceMaroniAlessandraZemaLuciaGazzanigaAndreaSezione di Tecnología e Legislazione Farmaceutiche “M. E. Sangalli”, Dipartimento di Scienze Farmaceutiche, Università degli Studi di Milano, 20133 Milano, Italy*Correspondence: marco.uboldi@unimi.it

The water-induced shape-memory response of pharmaceutical-grade poly(vinyl alcohol) (PVA) of different molecular weight was recently demonstrated [76,77]. Based on the ability of PVA-based items fabricated by fused deposition modeling (FDM) to take on a temporary shape and recover the original one upon contact with aqueous fluids at body temperature, 4D printing (i.e., 3D printing of objects that undergo changes in configuration upon exposure to an external non-mechanical *stimulus*) was proposed for the development of retentive systems intended for intragastric and intravesical drug delivery. Hot melt extrusion (HME) of rods to be wrapped around purposely developed templates was used for the rapid screening of different shapes potentially suitable for the site of retention. The temporary shape would allow the administration of the system, whereas the original bulky one would ensure its long-lasting residence in the target organ, by avoiding rapid emptying through the relevant opening. Following shape recovery, the system would ensure prolonged release of the conveyed drug and be safely eliminated by eroding/dissolving. Because the duration of release is a key issue for retentive drug delivery systems, the aim of the present work was to evaluate the application of permeable coatings based on insoluble film-forming polymers (e.g., Eudragit^®^ RL/Eudragit^®^ RS) to PVA devices as a possible approach to modulate the release rate without affecting the relevant shape-memory behavior.

Cylindrical rods of about 5 cm in length were prepared by HME (1.5 mm die, 215 °C, 20 rpm, 180 N·cm) starting from a PVA formulation (Gohsenol™ EG 48P plasticized with glycerol, 15% w/w on the dry polymer) containing 10% of a drug tracer. An ethanolic solution of Eudragit^®^ RS and Eudragit^®^ RL (3:1 w/w) containing 15% (w/w on the dry polymers) of triethyl citrate as the plasticizer was used as the coating formulation. A coating equipment was assembled, composed of: (i) a peristaltic pump (speed 10 rpm), (ii) a spray gun (nozzle 0.8 mm, atomizer 0.75 atm, pattern 1 atm) and (iii) an epicyclic planetary gearbox, in-house fabricated via FDM. The latter comprised 2 sets of gears, including the “sun” gear, directly moved by the rotor (2.3 rpm), and 4 satellite gears. The latter were in-built with the former and allowed positioning, along the gearbox height, of 4 samples at one time. The sun gear moved around its own axis only, while the satellites also rotated around the sun (i.e., revolution). In this way, the items to be coated were periodically exposed to the spray assembly, the rest of the rotation/revolution movement allowing for solvent evaporation. Through different process times, i.e., 4, 8 and 16 min, coated rods with increasing weight gain and coating thickness were obtained. After curing in a ventilated oven at 40 °C for 2 h, the samples were manually programmed to take on a temporary U-shape and quality of the film after this process was visually checked. The shape recovery behavior of the samples was not affected by the presence of the coating, at any thickness. On the other hand, the duration of release was enhanced as a function of the thickness of the applied film.

The results obtained proved the potential of the proposed approach, based on coating of shape-memory PVA-based systems fabricated by HME, for modulation of the drug release rate from retentive delivery systems.

**Acknowledgments:** No funds were received for covering the costs of this study.

**Conflicts of Interest:** The authors declare no conflict of interest.

### 3.25. Strategies to Improve the Solubility of Poorly Soluble Drugs by Soluplus^®^ Nanomicelles

CorsaroRobertaCurcioClaudiaCarboneClaudiaPignatelloRosario*Department of Drug Sciences, University of Catania, 95125 Catania, Italy*Correspondence: r.pignatello@unict.it

**Figure 20:**
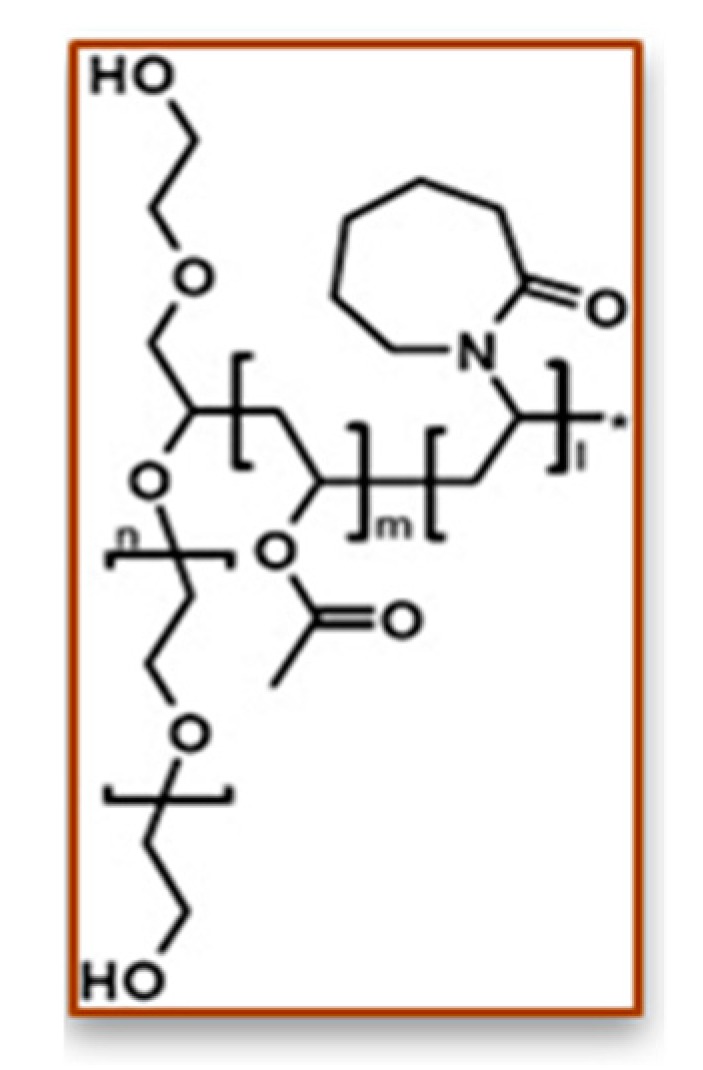
Structure of Soluplus^®^.

Soluplus^®^ (Figure 20) is a graft amphipathic copolymer consisting of polyvinyl caprolactam (57%), polyvinyl acetate (30%), and polyethylene glycol (13%) produced by BASF [78]. Soluplus^®^ exhibits amphipathic properties due to its hydrophilic and hydrophobic residues and it spontaneously forms spherical micelles in aqueous solution above a CMC of 7.6 mg/L.

Compared to conventional surfactants, Soluplus^®^ produces smaller spherical micelles (70–100 nm), with a hydrophobic core–hydrophilic shell structure that can help solubilizing poorly soluble compounds. The spherical structure of these nanomicelles reduces the free energy of the system and can aid for the production of highly water-soluble formulations [79,80,81,82,83].

The aim of this study was to prepare Soluplus^®^ micelles to enhance the solubility of three model APIs belonging to class II of the Biopharmaceutics Classification System (BCS), i.e., characterized by a poor solubility and a good permeability profile: ibuprofen (Ib), idebenone (Id) and miconazole (Mc).

Nanomicelles were produced by two alternative methods, namely direct dissolution of the drug in a 1.5 mM aqueous solution of Soluplus^®^ (dd) or by solvent evaporation–thin film hydration method (tf), in which the polymer and the drug were co-dissolved in acetone and evaporated to dryness, after which water was added under stirring at r.t., to produce the micelles at the wished polymer concentration.

The obtained nanomicelle suspensions were transparent, slightly opalescent when compared to water. No differences were observed between loaded and unloaded micelles in terms of mean size (Z-ave). Blank Soluplus^®^ micelles had an average size of 61.78 ± 1.61 nm and a polydispersity index (PDI) of 0.068 ± 0.022; the micelles loaded with the three APIs showed analogous values (Table 2).

**Figure 21:**
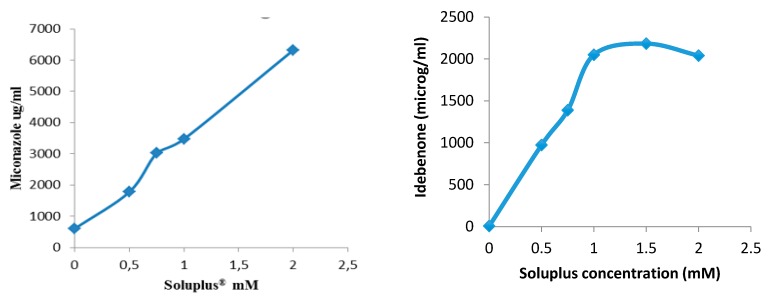
Solubility profiles of miconazole (left) and idebenone (right) as a function of Soluplus^®^ concentration.

Solubility of the tested APIs increased linearly with the concentration of Soluplus^®^, up to 290-fold for idebenone (Figure 21).

**Figure 22:**
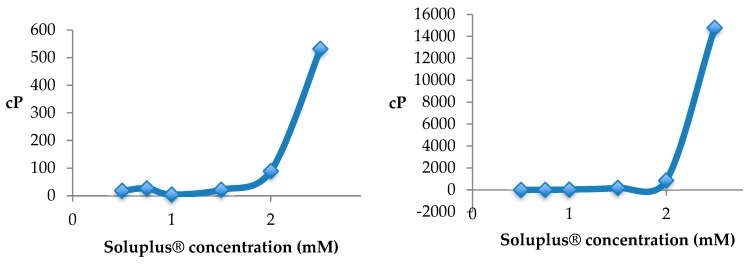
Viscosity of nanomicelle suspensions as a function of Soluplus^®^ concentration at 25 °C (left) and 35 °C (right).

Rheological studies (Figure 22) indicated that Soluplus^®^ micelles are suitable for ocular application, with a viscosity lower than 100 cP up to a 2 mM concentration, while at 35 °C (i.e., the temperature of corneal surface), they tend to become more viscous and bioadhesive.

In terms of Z-ave and PDI, the SNM-Id batches were stable at 4, 25 and 37 °C up to 6 months; SNM-Ib and SNM-Mc were stable for up to 3 months.

The drug-loaded nanomicelles can be easily sterilized by filtration through either PES or PTFE 0.22-µm membranes, without significant changes in the mean particle size.

Furthermore, loaded Soluplus^®^ nanomicelles suspensions were lyophilized, both in the absence or by addition of trehalose as a cryoprotectant (at 5.75 or 17.25%, w/v) and the resulting powder was easily redispersed with water, obtaining only a slight increase of Z-ave values, which however remained largely below 100 nm.

In summary, Soluplus^®^ micelles showed to be a valid mean to improve the solubility of BCS-class II drugs [83]. The micellar systems showed mean size highly suitable for ocular application, together with various technological features, such as possibility to be sterilized by membrane filtration and to be lyophilized without loss of integrity, which would depose for their potential use in pharmaceutical formulations.

**Acknowledgments:** BASF is gratefully acknowledged for gifting Soluplus^®^.

### 3.26. Developing a 3-Compartment Eye Flow Cell for Pre-Clinical Investigation

SapinoSimona*ChirioDanielaPeiraElenaGallarateMarinaDepartment of Drug Science and Technology, University of Torino, 10125 Torino, Italy*Correspondence: simona.sapino@unito.it

Pre-clinical pharmacologic studies for new preparations, aimed to investigate the mode of action, the effects, as well as ocular safety and pharmacokinetics of drugs and devices, have been so far dominated by the use of animal models. Despite their importance, presently many efforts are underway to reduce the number of animals used in testing, both to comply with ethical concerns and to reduce costs and time. Other findings of animal testing are the differences between animals and humans, the possible formation of antidrug antibodies and the variability of the experimental techniques used for each species.

In vitro models can offer a valid alternative to animal studies; among these, several have been established in the past few years to mimic different parts of the ocular system; anyway, little has been reported about in vitro model that accounts for the aqueous perfusion flow.

**Figure 23:**
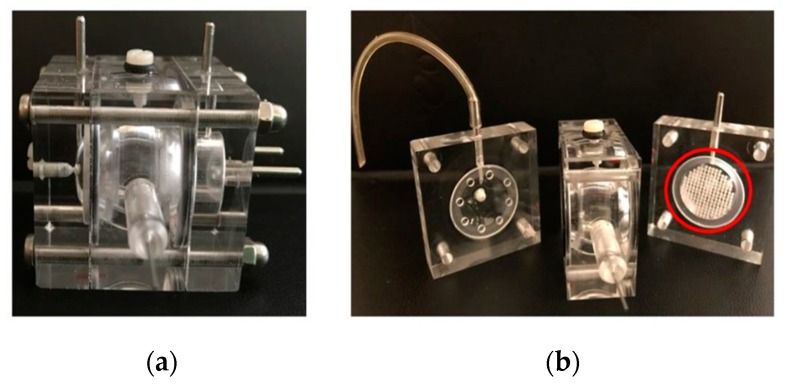
Ocular flow cell: (**a**) the three-compartments unit secured with four screws; (**b**) the red-circled semipermeable disk, possible support for retinal cells.

To address this gap, starting from the literature [84], we engineered a Plexiglas 3-compartment eye flow cell (Figure 23), mimicking the anterior and the posterior cavities of the eye. In addition, a semipermeable disk is placed vertically between the posterior section and a retro-retinal hollow unit that can be used as a support for retinal cell cultures to mimic the blood retinal barrier. An injection port is placed at the top of the vitreous cavity, while an aqueous inlet port connected to a dispensing pump, ensuring a perfusion flow rate of 2.0 µL/min, is placed in the vitreous cavity near the membrane barrier and an outlet port is placed in the anterior cavity to mimic the aqueous outflow in humans. The cell is filled with simulated vitreous (0.2% w/w agar/0.25% w/w hyaluronic acid).

Preliminary experiments were performed on dyes solutions to validate the model and then on (i) cefuroxime-loaded nanocomposite thermoreversible hydrogel and (ii) Avastin^®^ solution. In all experiment’s samples were injected into the posterior cavity while, at scheduled times, samples were collected from the anterior outlet port and then analyzed by HPLC to evaluate the concentration.

Although the model is still in an experimental stage, preliminary data showed this model to be a promising tool to obtain pre-clinical information about novel ophthalmic preparation namely to predict (1) dissolution/distribution (2) release profile and (3) clearance from the vitreous cavity of the candidate drug.

Further efforts must be focused towards providing this ocular cell with a posterior disk impregnated with Adult Retinal Pigment Epithelial cells (ARPE-19) mimicking the retinal pigment epithelium to create a more robust and effective cell-based platform for ophthalmic research.

**Acknowledgments:** This research was funded by Fondazione CRT-grant RF 2017.0820 and co-funded by MIUR, University of Turin-Fondi Ricerca Locale (ex–60%).

### 3.27. Is It Possible to Protect Liposomes Prepared by Ethanol Injection Upon Freeze-Drying

SelminFrancesca*FranzèSilviaRoccoPaoloMinghettiPaolaCilurzoFrancescoDepartment Pharmaceutical Sciences, Università degli Studi di Milano, via G. Colombo 71, 20133 Milan, Italy*Correspondence: francesca.selmin@unimi.it

Ethanol injection is an attractive technique for scaling-up liposome production. It is easily feasible at laboratory scale without sophisticated equipment and the method can be implemented at industrial scale by using crossflow or microfluidic devices, appositely developed [85]. However, the purification from residual solvent is often required. Lyophilization of the liposomal dispersion would avoid this step, increasing concomitantly the shelf-life of the product [86]. Nevertheless, the evaporation of ethanol, which does not freeze completely, may affect the quality of the freeze-dried product and therefore the reconstitution of the liposome dispersion.

This work aims to study the influence of the residual ethanol content on the feasibility of freeze-drying hydro-alcoholic liposomal dispersions. Liposomes composed of 1,2-dipalmitoyl-sn-glycero-3-phosphocholine (DPPC) and cholesterol (70:30 mol/mol) sizing 130 nm were prepared by ethanol injection and processed to have different final ethanol contents, namely 0.1, 1 and 6% v/v. Liposomes prepared by thin film hydration method were used as a negative control. The possible interactions with trehalose or saccharose (5:1 molar ratio), poly(vinyl pyrrolidone) K12 (PVP) (0.50 or 0.75%) and the binary mixtures were experimentally and computationally investigated.

All excipients were compatible with liposomes, even if PVP caused the formation of large aggregates after thawing and trehalose proved to be a better cryoprotectant than sucrose. This result agreed with the molecular dynamic simulations since both the free energy difference calculations and dipole alignment demonstrated that interactions with a DPPC bilayer were stronger for trehalose than sucrose.

Based on compatibility results, only hydro-alcoholic liposomal dispersions containing trehalose, PVP at 0.5% w/v or combination thereof were subject to thawing. The liposome characteristics were not altered upon freezing in agreement with the microregion entrapment theory. Indeed, upon freezing the ethanol remained uniformly entrapped as a liquid in the solid microregions allowing liposomes to reorganize without undergoing damages caused by ice crystals or cryo-concentration. Thus, the possible effect of protectants upon thawing was deepened on liposomes without ethanol. Trehalose and PVP at 0.5% m/v in mixture permitted to preserve liposome size distribution upon thawing.

After lyophilization, the moisture and the ethanol content were lower than 1% and negligible, respectively. However, the cake appearance was dependent of the ethanol content in the original dispersion. Up to 1%, the dried formulation was “blown out” of the vial (“product ejection”) because of the poor cohesion within the cake and solvent vapor pressure upon sublimation.

Upon reconstitution of freeze-dried products in the presence of a single protectant, the formation of too dispersed aggregates prevented DLS analysis. At ethanol contents lower that 1%, cakes presented an acceptable consistency and the reconstitution in water occurred within 30 min. However, liposomes freeze-dried in the presence of a single protectant showed an increased size (DH = 550 nm). Size comparable to that before drying (DH = 232 ± 15 nm) were obtained instead after reconstitution of formulation obtained using the binary mixture of excipients.

In conclusion, liposomes prepared by ethanol injection must be subjected unavoidably to mild processes of solvent evaporation before freeze-drying. A small residual ethanol content is allowed when a combination of trehalose and PVP is added to the formulation to preserve liposome integrity.

### 3.28. In Vivo Evidence for Novel Brain Targeted Peptide-Conjugated Nanomedicines

DuskeyJason Thomas^1^TosiGiovanni^1^*OddoneNatalia^1^OttonelliIlaria^1^VilellaAntonietta^2^KovachkaSandra^3^SpyrakisFrancesca^3^VandelliMaria Angela^1^RuoziBarbara^1^1Nanotech Lab, Te.Far.T.I., Dept. Life Sciences, University of Modena and Reggio Emilia, 41121 Modena, Italy2Department of Biomedical, Metabolic and Neural Sciences, University of Modena and Reggio Emilia, 41121 Modena, Italy3Department of Drug Science and Technology, University of Turin, 10124 Torino, Italy*Correspondence: Gtosi@unimore.it

The Central Nervous System (CNS) compartments remain one of the most difficult districts for drug delivery. This is due to the presence of Blood Brain Barrier (BBB) that hampers the passage of 90% of drugs, dramatically requiring improved strategies for successful non-invasive treatments. Here we describe for the first time the use of deltorphin-derived peptides to deliver biodegradable and biocompatible polymeric (i.e., poly-lactide-co-glycolide, PLGA) nanomedicines across the BBB. Opioid-derived peptides were covalently conjugated to furnish activated polymers, which were further used along with in conjunction with fluorescent tags to create targeted, trackable, nanoformulations. We report on the production, formulation set up, full physico-chemical nanoparticle characterization, and in vivo tests which generated clear proof of BBB-crossing and CNS-targeting by engineered nanomedicines opening the path to further applications for drug delivery and targeting in CNS disease models.

**Acknowledgments:** Supported by UNIMORE grant FAR (P.I. ZOLI), MAECI grant (P.I. Tosi, Nanomedicine for BBB-crossing in CNS oncologic pathologies). A.V. supported by a fellowship funded by the Dipartimento di Eccellenza Program 2018–2022, MIUR. 2018–2023 IMI EU Grants: Investigating Mechanisms and Models Predictive of Accessibility of Therapeutics (IM2PACT) Into the Brain. P.I. Zam Cader (UNIV OXFORD). 2019–2020 PROGETTI DI RICERCA SCIENTIFICA E TECNOLOGICA DI GRANDE RILEVANZA, Ministero degli Esteri, Progetti Italy-USA: Nanomedicine for BBB-crossing in CNS oncologic pathologies. PI. Prof G Tosi. The authors also gratefully acknowledge professional technical of CIGS staff (University of Modena and Reggio Emilia) for assistance in confocal and NMR analysis, the Centro di Competenza sul Calcolo Scientifico (C3S) of the University of Turin (c3s.unito.it) for providing the computational time and resources, and BiKi Technologies for providing the BiKi LiFe Sciences suite.

### 3.29. Synthesis and in Vitro Studies of HyDrO-DiAb Hydrogel Loaded with Green-Synthetized AgNPs for the Treatment of Diabetic Foot Ulcer

RuffoMariarosa^1^,^2^ParisiOrtensia Ilaria^1^,^2^AmoneFabio^2^MalivindiRocco^1^,^2^PezziVincenzo^1^,^2^TzanovTzanko^3^PuociFrancesco^1^,^2^1Department of Pharmacy, Health and Nutritional Sciences, University of Calabria, 87036 Rende (CS), Italy2Macrofarm s.r.l., c/o Department of Pharmacy, Health and Nutritional Sciences, University of Calabria, 87036 Arcavacata di Rende (CS), Italy3Molecular and Industrial Biotechnology group, Department of Chemical Engineering, Polytechnic University of Catalonia, Terrassa, 08018 Barcelona, Spain*Correspondence: Francesco.puoci@unical.it

Diabetic foot ulcerations (DFUs) represent one of the major complications for diabetic patients and for those patients which present neuropathy, peripheral vascular diseases and ischemia [87]. In healthy patients, wound healing is a dynamic, natural and regular biological process, in which different kind of cells, proteins and growth factors act to restore the structural integrity of injured skin [88]. In diabetic patients, the onset of sensor neuropathy prevents recognition of a foot ulcer and once it develops, the risk of infection increases dramatically. In in this kind of patients the normal response to bacterial infection, is missing. In the aim to control and treat DFUs, wound dressings able to absorb exudate, to prevent infections and to promote wound healing, are needed [89]. Chitosan (Chi) and Carboxymethylcellulose (CMC) are most common biodegradable and biocompatible polymers which could be used for the development of different modern wound dressings, either alone or as a part of multilayered wound dressings [90]. Several studies reported that silver nanoparticles (AgNPs) contribute to the wound healing process due to their antioxidant, antimicrobial and anti-inflammatory activity and to their simple incorporation in cotton fabric and dressings [91]. The aim of this work was to develop a biodegradable and biocompatible hydrogel composed by Chi and CMC (HyDrO-DiAb) loaded with AgNPs for the treatment of diabetic foot ulcer.

AgNPs were synthetized with a green synthetic approach using Olive Leaf dry extract (OLE), as a reducing agent, in a reaction with Silver Nitrate (AgNO_3_), to reduce Ag^+^ in Ag^0^. The synthesis reaction between OLE and AgNO_3_ was performed at room temperature, under stirring conditions and for 20 min. At the end of this time, the obtained nanoparticles were purified by centrifugation at 9000 rpm for 30 min and, then, washed thrice with distilled water, frozen, and lyophilized for further use. The obtained AgNPs were tested in terms of shelf stability and antioxidant activity, to evaluate, so, their possible use for the development of wound healing dressing. Once the shelf stability and the antioxidant activity were confirmed, AgNPs were loaded in HyDrO-DiAb hydrogel. The synthetized HyDrO-DiAb was characterized in terms of Water Content (%) and Moisture Retention Capacity (%) comparing the results with a hydrogel synthetized with only Chi and CMC. The obtained data evidence that are no statistical differences between HyDrO-DiAb and Chi-CMC hydrogel. Both maintain the same swelling behavior and water retention capacity. The in vitro cytotoxicity of HyDrO-DiAb was evaluated by MTT assay on HaCaT cell lines by exposing these cell lines to different concentrations of HyDrO-DiAb for 24 h. Results revealed that the cytotoxicity of sample was dose dependent and there is not cytotoxicity effect of HyDrO-DiAb sample, in the tested concentrations, since the cells growth inhibition rate was lower than 70%. In the aim to evaluate the release profile of AgNPs from HyDrO-DiAb, the tested sample was immersed for 8 h at 37°C in a PBS pH 8 and, at fixed time intervals, the absorbance of supernatant was measured at 430 nm. The obtained data show that the release profile of AgNPs was very fast, moreover, after 8 h, more than 90% of nanoparticles was released from the hydrogel. Finally, the wound healing ability of HyDrO-DiAb was evaluated by the cell scratch method on HaCaT cells, testing different concentrations of hydrogel for 24 h. The obtained results evidence that low concentrations of HyDrO-DiAb can in vitro close a wound and, so, silver nanoparticles used in this formulation could affect positively the wound healing process.

In summary, the performed studies evidence that the synthetized HyDrO-DiAb hydrogel may be potentially useful in wound management. These results point out the positive effect of the realized nanomaterials in speeding up the wound healing process, highlighting therefore, their potential application in wound repair.

**Acknowledgments:** This research was supported by MIUR (PON RI 2014–2020) in the framework of project Programma Operativo Nazionale FSE-FESR Ricerca e Innovazione 2014–2020, Azione I.1 “Dottorati innovativi a caratterizzazione industriale”—XXXIII Ciclo—A.A. 2017/2018.

### 3.30. Development of a New Theranostic Agent for Inflammatory Diseases

AilunoGiorgiaPastorinoSaraBaldassariSaraZuccariGuendalinaCaviglioliGabriele*DiFar, University of Genova, Viale Cembrano 4, 16148 Genova, Italy*Correspondence: caviglioli@difar.unige.it

Inflammation is a physiological response of our body, but it is also a pathological condition leading to the onset of different diseases like, among others, atherosclerosis, neurodegenerative disorders and cancer [92,93,94]. The onset of the inflammatory process involves several adhesion molecules: for example, the Vascular Cell Adhesion Molecule VCAM-1 is up-regulated during the initial step of the formation of the atherosclerotic plaque, allowing the accumulation of leukocytes in the expanding lesions. Recent studies have shown that VCAM-1 is also aberrantly expressed in different types of tumors, such as gastric, melanoma, ovarian, breast, and colorectal cancer [95]. Therefore, this adhesion molecule may be exploited as target for several inflammation-based diseases.

For these pathologies, a non-invasive technique suitable for both VCAM-1 overexpression imaging and targeted delivery of actives might be advantageous for theragnostic purposes.

**Figure 24:**
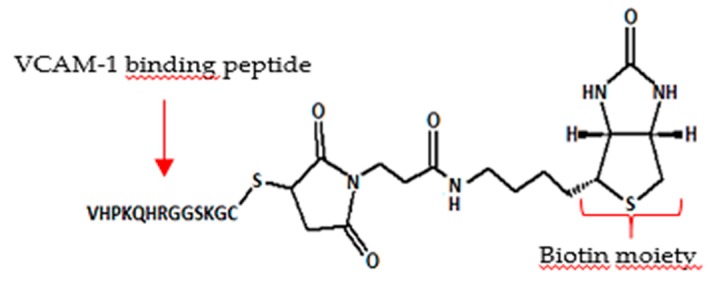
NAMP structure.

**Figure 25:**
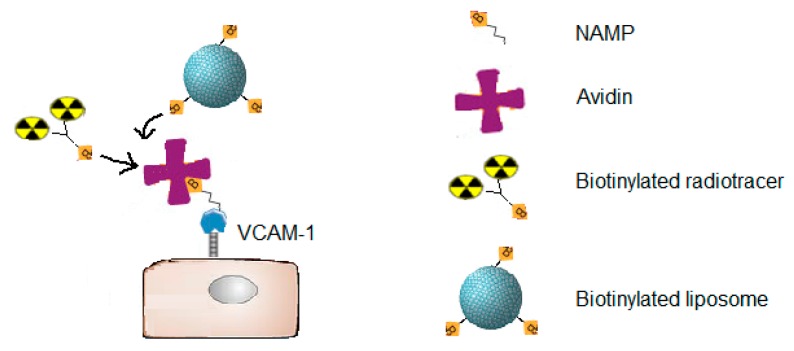
Three-step theragnostic system.

To this aim, we have developed a new molecule (NAMP, Figure 24), based on a biotin derivative linked to a VCAM-1 binding peptide. By exploiting the well-known biotin/avidin affinity, this conjugate might be employed in a sequential treatment including avidin and a biotinylated tracer radiolabeled with ^68^Ga, with the aim of obtaining a PET imaging of the site overexpressing VCAM-1. The conjugate has also been envisaged as pretargeting agent for biotinylated nanocarriers, such as liposomes, to deliver drugs to the inflammation sites (Figure 25).

The VCAM-1 binding peptide that is included in NAMP structure is a 13 amino acid peptide in which seven amino acids have sequence homology with the alpha-chain of integrin VLA-4, the natural ligand of VCAM-1, while the other six are introduced as a linker sequence. By Orbitrap-MS we confirmed NAMP molecular structure, and in particular we verified that the peptide was linked to the biotinylated moiety through the carboxy-terminal cysteine.

For the imaging application, we selected a double-chelating PET radiotracer which can be labeled with up to two radionuclides, thus increasing the signal deriving from the target and, consequently, the potentiality of the diagnostic approach.

The three-step pre-targeting system was tested in vitro on HUVEC cells activated with TNF-alpha and subsequently incubated first with NAMP, then with avidin and finally with ^68^Ga-radiotracer. The difference in radiosignal intensity shown by the activated cells compared to inactivated (control) cells was impressive.

For the therapeutic application we selected, as nanocarrier, liposomes, because of their biocompatibility and their similarity with cell membranes. Biotinylated stealth liposomes were prepared with the well-known hydration method of a lipid film, followed by extrusion and purification by gel filtration. Liposome size (161 ± 12 nm, polydispersity index between 0.04 and 0.1) was determined through photon correlation spectroscopy, while the zeta potential (−19.1 ± 0.6 mV) was measured by micro-capillary electrophoresis. The concentration of the liposomal suspension, determined through a Zetasizer Ultra, was 114 × 10^5^ particles/mL. After two-week storage at 4 °C, the liposomes did not show any significant difference in the dimensional or surface electrical characteristics.

A liposomal formulation including CM-DiI fluorescent dye was tested in vitro on human cancer cell cultures, applying the NAMP-avidin-liposomes protocol, and by live confocal fluorescence microscopy a preferential interaction of the biotinylated liposomes with the tumoral cells expressing VCAM-1 was observed.

In conclusion, NAMP could be exploited as a theragnostic tool for targeting VCAM-1.

**Conflicts of Interest:** The authors declare no conflict of interest.

## 4. Conclusions

The overall goal of 2019 CRS Italy Chapter Workshop was to focus the attention of researchers from academia and industry upon the relevance of developing innovative strategies for the controlled delivery and targeting of drugs and health-related products, such as medical devices, food supplements, and even cosmetics, keeping well in mind the real therapeutic and clinical needs. In the meantime, each novel technological platform must undertake as soon as possible a rigorous assessment in pre-clinical and clinical models.

The organizations believe that the merging of knowledge and in-field expertise from the invited speakers has given to the attendants many prompts and much advice to speed up and improve the overall quality of Italian academic research in controlled release technology.

## Figures and Tables

**Table 1:** DSC data.

Sample	Tg/°C	Tm/°C
PLGA	47.6 ± 0.7	-
F68 + F127	48.3 ± 0.4	54.1 ± 0.4
HA2	-	51.9 ± 0.9
HA8	-	52.0 ± 0.7

**Table 2:** Size analysis (Z-Ave), polydispersity index (PDI) and Zeta potential (ZP) values of drug-loaded Soluplus^®^ nanomicelles produced by the direct dissolution method (dd) or by thin film hydration (tf).

Formulation	Size Analysis			PDI	ZP
	Z-Ave	PK1	Area %		
**SNM-Id^dd^**	59.00 ± 1.028	63.06 ± 1.145	100	0.053 ± 0.011	−2.98
**SNM-Id^tf^**	60.60 ± 1.385	65.25 ± 3.108	100	0.056 ± 0.038	−1.23
**SNM-Ib^dd^**	50.20 ± 0.1450	54.22 ± 1.095	99.5	0.164 ± 0.022	−2.43
**SNM-Ib^tf^**	59.05 ± 0.9603	64.00 ± 0.6731	100	0.100 ± 0.053	−4.12
**SNM-Mc^dd^**	55.62 ± 0.0396	63.45 ± 3.265	98.8	0.189 ± 0.008	0.481
**SNM-Mc^tf^**	56.42 ± 3.5370	55.77 ± 4.956	99.9	0.156 ± 0.030	0.7896
